# A Randomised Controlled Trial of Consent Procedures for the Use of Residual Tissues for Medical Research: Preferences of and Implications for Patients, Research and Clinical Practice

**DOI:** 10.1371/journal.pone.0152509

**Published:** 2016-03-30

**Authors:** S. Rebers, E. Vermeulen, A. P. Brandenburg, T. J. Stoof, B. Zupan-Kajcovski, W. J. W. Bos, M. J. Jonker, C. J. Bax, W. J. van Driel, V. J. Verwaal, M. W. van den Brekel, J. C. Grutters, R. A. Tupker, L. Plusjé, R. de Bree, J. H. Schagen van Leeuwen, E. G. J. Vermeulen, R. A. de Leeuw, R. M. Brohet, N. K. Aaronson, F. E. Van Leeuwen, M. K. Schmidt

**Affiliations:** 1 Division of Psychosocial Research and Epidemiology, The Netherlands Cancer Institute, Amsterdam, The Netherlands; 2 Department of Dermatology, VU University Medical Center, Amsterdam, The Netherlands; 3 Department of Dermatology, The Netherlands Cancer Institute, Amsterdam, The Netherlands; 4 Department of Internal Medicine, St. Antonius Hospital, Nieuwegein, The Netherlands; 5 Department of Dermatology, Spaarne Gasthuis location Haarlem Zuid, Haarlem, The Netherlands; 6 Department of Obstetrics, VU University Medical Center, Amsterdam, The Netherlands; 7 Department of Gynaecology, The Netherlands Cancer Institute, Amsterdam, The Netherlands; 8 Department of Surgical Oncology, The Netherlands Cancer Institute, Amsterdam, The Netherlands; 9 Department of Head and Neck Oncology and Surgery, The Netherlands Cancer Institute, Amsterdam, The Netherlands; 10 Department Pulmonology, St. Antonius Hospital, Nieuwegein, The Netherlands; 11 Division of Heart and Lungs, University Medical Centre Utrecht, Utrecht, The Netherlands; 12 Department of Dermatology, St. Antonius Hospital, Nieuwegein, The Netherlands; 13 Department of Dermatology, Rode Kruis Hospital, Beverwijk, The Netherlands; 14 Department of Otolaryngology-Head and Neck Surgery, VU University Medical Center, Amsterdam, The Netherlands; 15 Department of Gynaecology, St. Antonius Hospital, Nieuwegein, The Netherlands; 16 Department of Surgery, Spaarne Gasthuis location Haarlem Zuid, Haarlem, The Netherlands; 17 Athena Institute for transdisciplinary research, VU University Medical Center, Amsterdam, The Netherlands; 18 Research Center Linnaeus Institute, Scientific Department, Spaarne Gasthuis, Hoofddorp, The Netherlands; 19 Division of Molecular Pathology, The Netherlands Cancer Institute, Amsterdam, The Netherlands; National Cancer Centre Singapore, SINGAPORE

## Abstract

**Background:**

Despite much debate, there is little evidence on consequences of consent procedures for residual tissue use. Here, we investigated these consequences for the availability of residual tissue for medical research, clinical practice, and patient informedness.

**Methods:**

We conducted a randomised clinical trial with three arms in six hospitals. Participants, patients from whom tissue had been removed for diagnosis or treatment, were randomised to one of three arms: informed consent, an opt-out procedure with active information provision (opt-out plus), and an opt-out procedure without active information provision. Participants received a questionnaire six weeks post-intervention; a subsample of respondents was interviewed. Health care providers completed a pre- and post-intervention questionnaire. We assessed percentage of residual tissue samples available for medical research, and patient and health care provider satisfaction and preference. Health care providers and outcome assessors could not be blinded.

**Results:**

We randomised 1,319 patients, 440 in the informed consent, 434 in the opt-out plus, and 445 in the opt-out arm; respectively 60.7%, 100%, and 99.8% of patients’ tissue samples could be used for medical research. Of the questionnaire respondents (N = 224, 207, and 214 in the informed consent, opt-out plus, and opt-out arms), 71%, 69%, and 31%, respectively, indicated being (very) well informed. By questionnaire, the majority (53%) indicated a preference for informed consent, whereas by interview, most indicated a preference for opt-out plus (37%). Health care providers (N = 35) were more likely to be (very) satisfied with opt-out plus than with informed consent (p = 0.002) or opt-out (p = 0.039); the majority (66%) preferred opt-out plus.

**Conclusion:**

We conclude that opt-out with information (opt-out plus) is the best choice to balance the consequences for medical research, patients, and clinical practice, and is therefore the most optimal consent procedure for residual tissue use in Dutch hospitals.

**Trial Registration:**

Dutch Trial Register NTR2982

## Introduction

Human tissue, stored after clinical procedures, e.g. after histopathological examination of a surgical specimen, is an important resource for medical research [1]. In the U.S., there is discussion whether patients’ consent should be asked once or for each study separately [2]. In Europe, the discussion centres around informed consent versus opt-out consent regimens. Dutch hospitals use an opt-out procedure for the use of these residual tissues. However, both the proposed Dutch Control of Human Tissue Act and the proposed European Commission’s General Data Protection Regulation may change the current system to one in which patients’ explicit informed consent is necessary for the pseudonymised use of residual tissue and accompanying data [3, 4]. It is uncertain what the consequences of a change to a more restrictive consent regimen would be [5, 6]. Consequences may be expected for medical research quality, patient satisfaction and informedness, and daily clinical practice. However, little research has been conducted on these consequences.

Medical research might be compromised in an informed consent procedure, because a large proportion of patients may not actively provide their consent [7, 8]. This was observed in a small study of cancer patients [9]. It has been suggested that the main reason for not completing a consent form is not unwillingness, but rather nonchalance, or lack of time [10]. Further, consenting patients may not be representative of the total patient population, because certain patient groups may be less likely to consent than others. Such bias has indeed been found for biobanks [11] and observational studies [12–14]; moreover the direction of the bias differs between studies [15].

Patients’ satisfaction might be compromised in an opt-out procedure because patients commonly receive scarce information about residual tissue use [16]. However, it has been suggested that an opt-out procedure could be acceptable when information is provided actively [17, 18]. Previously, we developed an intermediate procedure, ‘opt-out plus’ [16], which is an opt-out procedure with active information provision. We found that satisfaction with the received information was high both in patients who experienced an informed consent and an opt-out plus procedure, and that the majority of cancer patients preferred opt-out plus [9]. These findings, however, have not been confirmed in a more diverse and larger patient sample. We are unaware of any studies on health care providers’ (HCPs) experiences with different consent procedures for residual tissue use.

In this study we investigate the consequences of three consent procedures, informed consent, opt-out and opt-out plus, on consent rates, patients’ information appreciation, and their awareness of the potential storage of residual tissue. Further, we investigate patients’ preferences for consent procedures, and HCPs’ satisfaction with, and preferences for, the three procedures.

## Methods

In this randomised controlled trial, we randomised patients to one of three arms: informed consent, opt-out plus, or opt-out. In the opt-out group, standard care and control arm, patients did not receive verbal information about residual tissue use. Some hospitals provided information as part of a general hospital information leaflet. In the informed consent group, HCPs informed patients verbally about residual tissue use. This was estimated to take one minute to provide. The patient then received a leaflet specifically about residual tissue use, which contained five pages of information, an informed consent form, and a stamped return envelope. The opt-out plus procedure was similar to the informed consent procedure, except that the leaflet contained an opt-out form that patients only needed to return when they did not want their residual tissue to be used in medical research. An (updated) version of the brochure used in the opt-out plus procedure can be found online [19].

Patients were unaware of the study and randomisation at the moment of inclusion. We considered it necessary to postpone the (written) informed consent procedure, because our earlier studies showed it confused patients to be asked informed consent for a study in which they would be randomised to a consent procedure [9]. Further, asking consent would likely have caused bias in our outcome data, especially regarding the number of patients opting in or out of residual tissue use, because only patients interested and willing to participate in medical research would be enrolled. The IRB of the Netherlands Cancer Institute approved the trial, including this procedure.

Six Dutch hospitals included patients in the trial. Patients were eligible for inclusion if their tissue had been or was planned to be excised either for diagnostic or treatment purposes, they were between 18 and 80 years of age, and if they were told whether they had a malignant or benign disease. Patients were excluded if they did not speak and/or read the Dutch language, as were patients who had already been asked for informed consent for a *de novo* biobank.

Patients from whom the following tissue types had been (or were planned to be) excised were included: dermatological, colorectal, gynaecological, otolaryngological, urological, and pulmonal. Patients who had given blood samples were also included. We tried to ensure that for all tissue sites, both patients with benign and malignant disease were included (e.g. both skin cancer and eczema).

We sent a questionnaire to all patients six weeks after randomisation. Non-respondents received a written reminder after three weeks, and a telephone reminder after six weeks. AB and SR interviewed a subset of patients by telephone approximately one week after they returned the questionnaire (see S1 Methods). The HCPs received a short questionnaire about their experiences after patient inclusion had finished.

The randomisation was stratified by hospital, whether the tissue was malignant or benign, and tissue site. We randomised using envelopes. Randomisation was conducted using block randomisation in blocks of nine. HCPs and outcome assessors could not be blinded.

### Statistical analysis

Group comparisons were performed by parametric or non-parametric statistical tests, as applicable and indicated in the tables. For the multivariable models, we used logistic regression analyses. Independent variables were included in a model if their p-value was below 0.1 in a univariable analysis, but removed from the multivariable model if they were non-significant at the 0.1-level. In case of categorical variables with more than two categories (e.g. educational level) we used the p-value of the overall variable to determine whether or not to include them. A two-sided p-value below 0.05 was considered statistically significant. We performed all analyses using SPSS 22.

## Results

### Patient participation and characteristics

**Fig 1:**
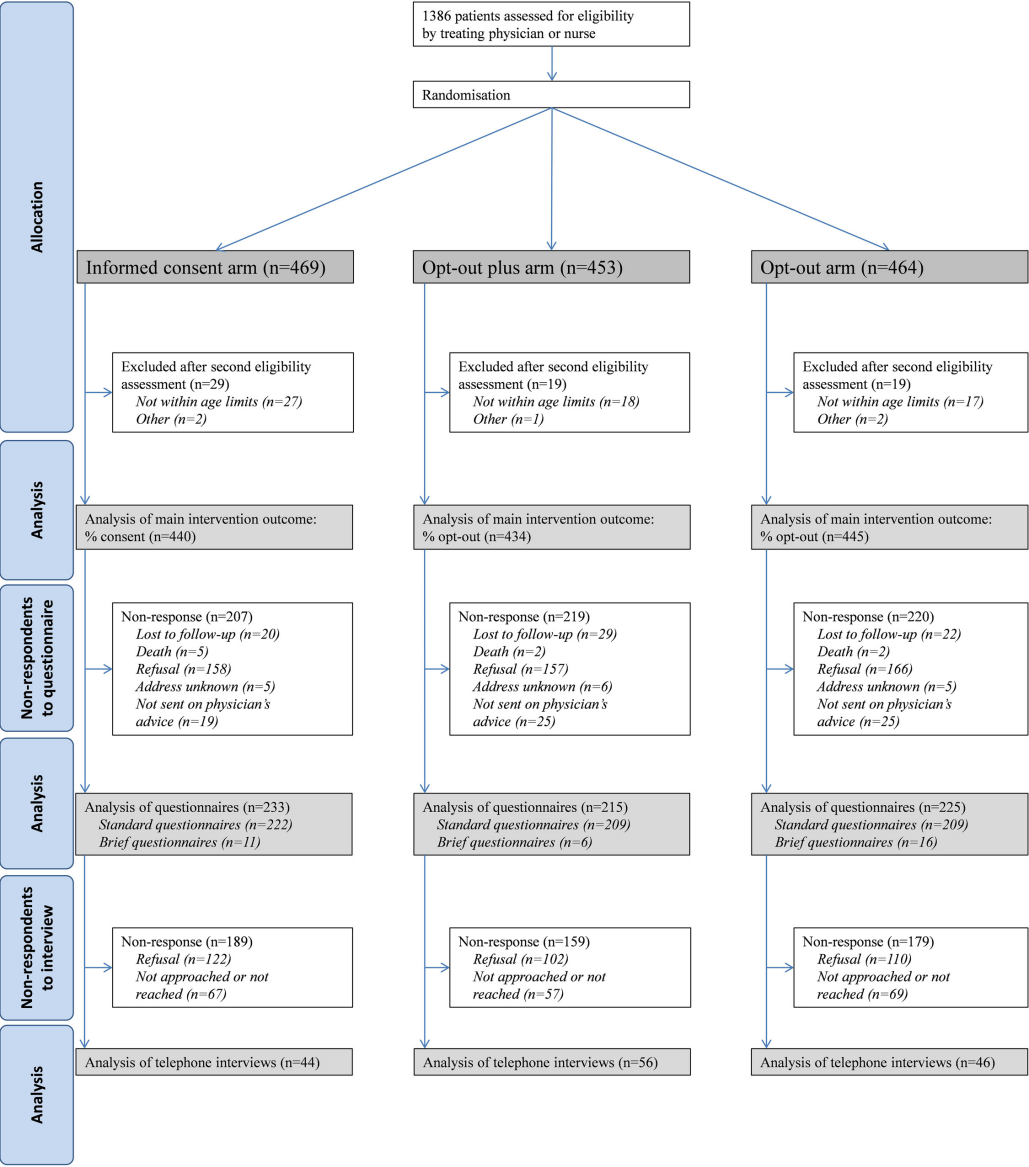
Flow-chart of the study.

We randomised 1,319 eligible patients, 440 to the informed consent, 434 to the opt-out plus, and 445 to the opt-out arm (Fig 1). In total 673 patients (51%) completed the questionnaire (hereafter: respondents). We conducted interviews with 146 respondents.

The majority of patients was female (61%), between 61 and 80 years of age (47%), and was treated in an academic hospital (62%). Characteristics of all patients and in subgroups, including differences between the total patient sample, questionnaire respondents, and interviewees, can be found in Table 1 and S1 Table, respectively. Patient characteristics per arm are provided in S2 Table.

**Table 1:** Characteristics of patients in the intervention study.

	All patients (N = 1319)	Respondents (no interview) (N = 527)
	%	%
**Intervention arm**		
Informed consent	33	36
Opt-out plus	33	30
Opt-out	34	34
**Sex**		
Male	39	40
Female	61	60
**Educational level (questionnaire data)**		
Low	9	18
Intermediate	22	41
High	16	31
Missing	53	10
**Age (years)**		
18–40	19	14
41–60	32	36
61–80	47	49
Missing	2	2
**Academic vs non-academic hospital**		
Academic hospital	62	55
Non-academic hospital	38	45
**Procedure**		
Excision	49	49
Biopsy or puncture	21	20
Blood withdrawal	22	22
Other	6	7
Missing	2	2
**Benign or malignant disease**		
Malignant disease	54	56
Benign disease	44	43
Unknown	1	1
**Tissue site**		
Dermatological	45	48
Otolaryngological	9	7
Gastroenterological	8	9
Pulmonal	4	4
Haematological	22	22
Gynaecological	12	11

### Medical research consequences

Based on the questionnaire (n = 673), 95% of respondents indicated that their tissue could be used for medical research, 2% that it could not be used, and 3% that they did not know. Interviewees all indicated their residual tissue could be used.

Significantly fewer patients in the informed consent arm consented to the use of their residual tissue, as compared to the opt-out plus and opt-out arms (Table 2). Specifically, none of the patients in the opt-out procedure objected to the use of their residual tissue, i.e. 100% consented. In the opt-out plus procedure, one of 434 patients objected to this use, i.e. 99.8% consented. In the informed consent arm, one patient actively refused consent, and 172 of 440 passively gave no consent as they did not return the consent form; i.e. only 60.7% of tissues could be used for medical research.

**Table 2:** Consent and experiences in/with the three trial arms.

	N	Trial arm			Informed consent vs. opt-out plus	Informed consent vs. opt-out	Opt-out plus vs. opt-out
		Informed consent	Opt-out plus	Opt-out			
**Consent**						P-value*	
Tissue availability, %	1319	60.7	99.8	100	<0.0001	<0.0001	0.311
**Patients’ experiences**						P-value*	
Proportion (very) well informed in trial arm, %	645	71	69	31	0.522	<0.0001	<0.0001
Proportion aware of storage in trial arm, %	666	71	71	30	0.876	<0.001	<0.001
Number of four knowledge questions correctly answered, mean (SD)	567	3.0 (0.9)	2.9 (1.0)	2.7 (1.1)	0.479	0.014	0.079
**Health care providers’ experiences**						P-value*	
Proportion (very) satisfied with procedure, %	31^A^	48	90	69	0.001	0.083	0.020
Proportion indicating procedure never interfered with clinical information, %	32^B^	62	69	91	0.083	0.005	0.014
Time spent informing patients and answering questions, mean (SD, minutes)^C^	28	3.0 (2.4)	1.7 (1.5)	-	0.005	-	-

^A^ N for opt-out arm 32

^B^ N for opt-out arm 29

^C^ See S1 Methods

*Mann-Whitney U-test/Wilcoxon signed ranks test for proportional variables, T-tests (paired or unpaired as appropriate) for continuous variables.

Compared to patients with a low educational level, patients with an intermediate/high educational level were less likely to return the consent form (Table 3; S3 Table). In the model based on intervention data, we found that older patients and patients treated in non-academic hospitals were more likely to return the consent form. All variables of the multivariable model based on data of all patients in the informed consent arm were non-significant in the model using only data of patients who returned the questionnaire, possibly because those who returned the questionnaire were more likely to return the consent form as well. Consent form return in this subsample was 73% (compared to 61% in the whole group). In the questionnaire data based model, patients who indicated better physical functioning were more likely to return the consent form.

**Table 3:** Determinants of returning a consent form in the informed consent arm*.

	**Multivariate model on intervention data including all patients in the informed consent arm (N = 424)**	
	**OR (95% CI)**	**P-value**
**Age (years)**	1.02 (1.01–1.04)	0.001
**Hospital type**		
Academic	Reference	
Non-academic	2.11 (1.29–3.48)	0.003
**Tissue site**		*0*.*003*
Dermatological	Reference	
Otolaryngological	0.65 (0.30–1.41)	0.279
Gastroenterological	0.36 (0.17–0.76)	0.007
Pulmonal	0.18 (0.06–0.51)	0.002
Haematological	0.54 (0.30–0.96)	0.037
Gynaecological	0.48 (0.25–0.93)	0.029
**Percentage highly educated individuals in respondent’s zip code area**^A^	0.98 (0.96–1.00)	0.060
	**Multivariate model on questionnaire data including only the patients in the informed consent arm who returned the questionnaire**^B^ **(N = 193)**	
	OR (95% CI)	P-value
**Educational level**^C^		*0*.*060*
Low	Reference	
Intermediate	0.25(0.08–0.78)	0.018
High	0.31 (0.09–1.01)	0.052
**Physical functioning (SF-12 subscale)**^D^	1.28 (1.01–1.62)	0.042

See also S1 Methods for background information on questionnaire and definitions of variables. OR = Odds Ratio, CI = Confidence Interval

* Results of logistic regression analyses.

^A^ CBS Netherlands, based on 4 digits

^B^ all of the above variables turned out non-significant in the model on questionnaire data

^C^ as indicated in the questionnaire. Respondents indicating a ‘different’ educational level were not taken into account in this analysis. If they are taken into account, the p-value of physical functioning is reduced to 0.073

^D^ a higher score indicates better physical functioning

### The patients’ perspective

Respondents in the informed consent and opt-out plus arms indicated significantly more often that they were (very) well informed about residual tissue use (69% and 71%) versus patients in the opt-out arm (31%) (both comparisons p<0.001, Table 2). Similarly, in both the informed consent and opt-out plus arms 71% of respondents were aware of potential tissue storage compared to 30% in the opt-out arm (both comparisons p<0.001).

Out of four questions used to assess respondents’ knowledge about residual tissue use, respondents in the informed consent arm had similar scores to those in the opt-out-plus arm (p = 0.479), but answered statistically significant more questions about residual tissue use correctly than those in the opt-out arm (p = 0.014; Table 2).

Significantly more respondents indicated in the questionnaire that they preferred an informed consent procedure for residual tissue use (53%), as compared to opt-out plus (31%) and opt-out (11%) (all p-values<0.001; Table 4). Six percent indicated they did not know or had no preference. During the interviews, significantly more patients preferred opt out-plus (37%) than opt-out (17%) or informed consent (12%) (p = 0.001; Table 4).

**Table 4:** Preferences for one of three procedures of patients and HCPs.

		Preference for informed consent	Preference for opt-out plus	Preference for opt-out	No (clear) preference	Informed consent vs. opt-out plus	Informed consent vs. opt-out	Opt-out plus vs. opt-out
	N	%	%	%	%		P-value*	
	**Patient preferences**							
In questionnaire	660	53	31	11	6	<0.0001	<0.0001	<0.0001
In interview^A^	146	17	37	12	34^A^	0.001	0.217	<0.0001
	**Patient preferences in interview grouped by preferences in questionnaire**^B^							
Preference for informed consent in questionnaire	65	26	31	5	39	0.622	0.002	0.39*10^−3^
Preference for opt-out plus in questionnaire	56	13	45	14	29	0.001	0.796	0.003
Preference for opt-out in questionnaire	20	5	45	20	30	0.011	0.180	0.166
No preference in questionnaire	4	0	0	50	50	n/a	n/a	n/a
	**HCP preference**							
In questionnaire	32^C^	9	66	25	n/a	0.23*10^−3^	0.132	0.016

^A^ 32% did not indicate a (clear) preference for a consent modality or felt the hospital should not let patients decide, in 2% of interviews the consent modality was not discussed, and 1% indicated a preference for informed consent but also indicated the hospital should not inform patients

^B^ 145 interviewees responded to the preference question in the questionnaire

^C^ In total we received 35 questionnaires, in 32 questionnaires the preference was indicated

* Results of Chi-square tests

**Fig 2:**
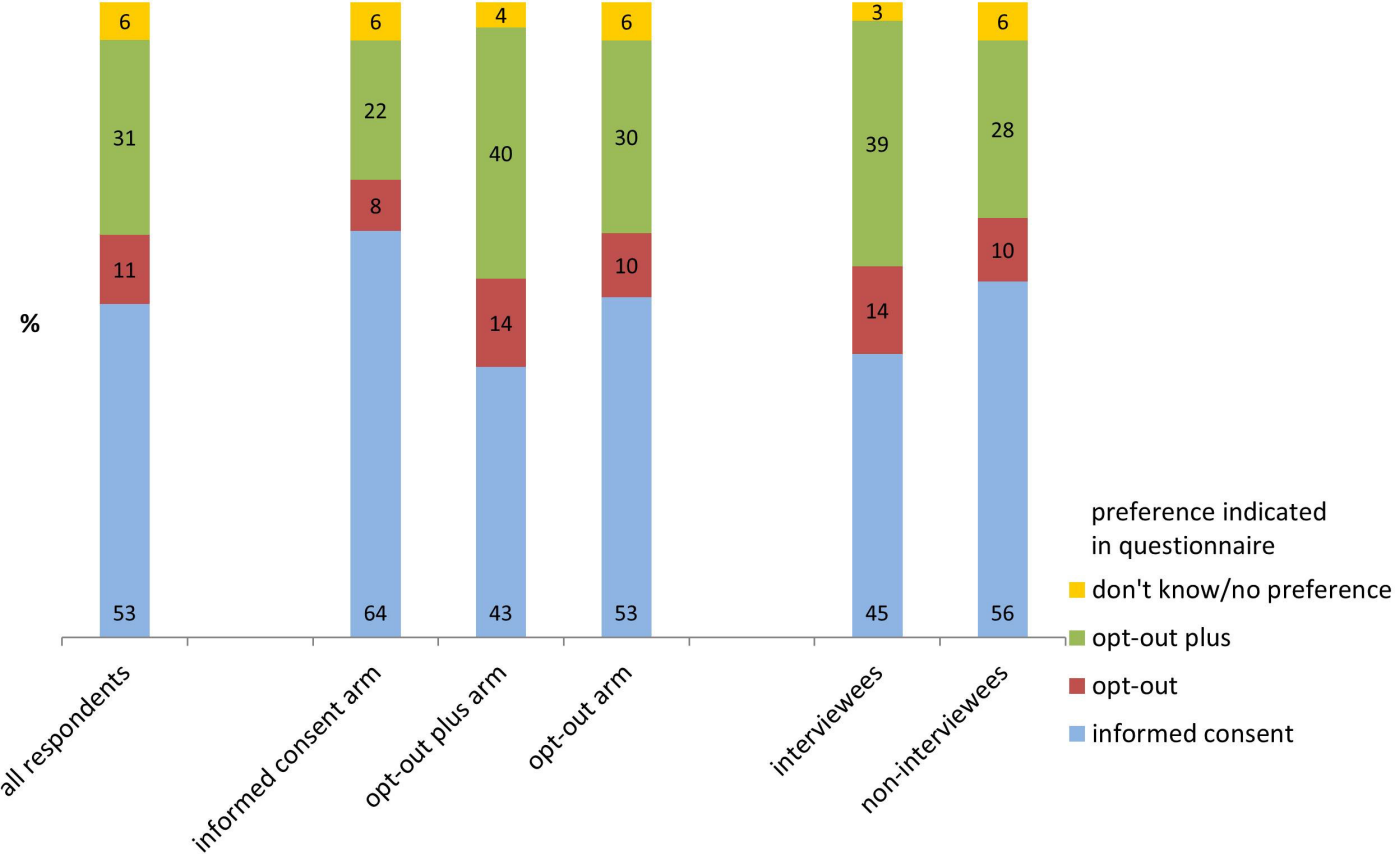
Consent procedure preferences in the questionnaire for all respondents, respondents in the three intervention arms, and interviewees and non-interviewees*. *The results of logistic regression analyses testing the statistical significance of the differences between these groups are reported in S4 Table.

Respondents who preferred opt-out plus in the questionnaire were more likely to be interviewed (Fig 2 and S4 Table). Moreover, whether a patient changed preference during the interview depended on their intervention arm. Of those patients indicating a preference for opt-out plus in the questionnaire, 45% did not change their preference in the interview, and 13% changed towards informed consent. Of those respondents indicating a preference for informed consent in the questionnaire, 26% did not change their mind in the interview, but 31% changed their preference to opt-out plus (Fig 2 and S4 Table).

Interestingly, the percentages of respondents preferring the three consent procedures differed as a function of the trial arm. Respondents in the informed consent arm were more likely to prefer informed consent (64%) than respondents in the opt-out plus arm (43%) (p = 0.006). Likewise, respondents in the opt-out plus arm were more likely to prefer opt-out plus (40%) than respondents in the informed consent arm (22%) (p<0.001) (Fig 2 and S4 Table).

### The HCPs’ perspective

In total 59 HCPs (83% physicians, 8% nurse practitioners, and 8% nurses) participated in the study. On average they included 22 patients (SD = 31.1). The post-intervention questionnaire was filled in and returned by 35 (59% response).

Significantly more HCPs were (very) satisfied with the opt-out plus procedure (90%) than with the opt-out procedure (69%) and the informed consent procedure (48%) (p = 0.020 and p = 0.001, respectively; Table 3). The HCPs indicated that the opt-out procedure interfered significantly less often with giving clinical information (91%) than either the informed consent (69%) or the opt-out plus procedure (62%; p = 0.005 and p = 0.014, respectively; Table 3). On average HCPs spent 3.0 minutes (SD = 2.4) informing patients about residual tissue use and answering their questions in the informed consent condition, while they spent 1.7 minutes (SD = 1.5) in the opt-out plus condition (p = 0.005; Table 3). HCPs indicated a clear preference for the opt-out plus procedure (66%) versus opt-out (25%) and informed consent 9% (p = 0.016 and p<0.001, respectively; Table 4).

## Discussion

With this study, we investigated the consequences of three consent procedures for the availability and representativeness of residual tissue for medical research, patient and HCP preferences, and HCP satisfaction with each procedure. We found that 1) the opt-out and opt-out plus procedures resulted in high availability of bias-free tissue for medical research, 2) patients were well-informed in the informed consent and opt-out plus procedures, 3) patients indicated a preference for informed consent in the questionnaire, but a preference for opt-out plus in the interviews, 4) HCPs were most satisfied with the opt-out plus procedure, and 5) HCPs preferred the opt-out plus procedure (Table 5).

**Table 5:** Qualitative summary of outcomes of the three consent procedures.

	Informed consent	Opt-out plus	Opt-out
**Medical research consequences**			
Tissue availability (consent rates)	**-**	**+**	**+**
Bias	**-**	**+**	**+**
**Patient consequences**			
Perceived informedness (satisfaction)	**+**	**+**	**-**
Awareness of residual tissue storage	**+**	**+**	**-**
Knowledge^B^	**+**	**+**	**+**
Conform patient preference^A^	**+**	**+**	**-**
**HCP consequences**			
HCP satisfaction	**-**	**+**	**+**
Conform HCP preference	**-**	**+**	**-**
No interference with clinical information^B^	**-**	**-**	**+**
Time investment^B^	**-**	**-**	**+**

^A^ Based on both questionnaires (most preference for informed consent) and interviews (most preference for opt-out plus)

^B^ Secondary outcome

HCP = Health Care Providers

A limitation of our study is that subgroups were small, making it impossible to directly investigate whether biases based on for instance tumour morphology exist in the informed consent arm in our sample. Importantly, it is very likely that the biases we found, especially age, educational level and hospital type, correlate strongly with disease characteristics, and thus influence the external validity of medical studies if informed consent would be implemented. Another limitation is that although we included patients with a wide variety of diseases, our sample is not a random sample of the patient population. Further, questionnaire respondents and interviewees were not a random subsample. For instance, respondents and interviewees were somewhat older than the patients in our sample, and we do not know the extent to which this may have affected our outcomes. However, it is unlikely that the difference found in preferences between questionnaire respondents and interviewees was caused by bias, because patients with the overrepresented patient characteristics in the interviewee subsample were not more likely to prefer an opt-out procedure (data not shown).

In this study, we did not thoroughly investigate which factors determined the consent procedure preferences indicated by patients. Therefore, we cannot fully explain why respondents indicated different preferences in the questionnaire versus the interview. However, part of the observed difference may be explained by selection bias: respondents who indicated a preference for opt-out plus in the questionnaire were more likely to be interviewed. Importantly, an additional explanation could be that during interviews, patients received more, or better tailored, information, which may have led them to change their opinion. Residual tissue use is an uncommon topic for most patients, and it is therefore likely that differences in the content, complexity, or amount of information given influence patients’ opinions. This could explain why we observed that more interviewees changed their preference for informed consent in the questionnaire to opt-out plus in the interview than the other way around. Such an effect has been reported previously by Lewis et al. [20], who found that individuals who participated in focus groups were more accepting of less restrictive consent models than those who expressed their opinions via a questionnaire.

Although many HCPs filled in our questionnaire, we only included HCPs who were willing to spend time informing patients about residual tissue, which may have led to a larger proportion favouring informed consent or opt-out plus. A surprising finding is that the percentage of HCPs indicating that opt-out never interfered with giving clinical information was not 100%. A possible explanation is that some felt that the small administrative task related to the trial interfered with giving clinical information. This task was similar for all procedures, and is therefore unlikely to have biased our results.

Our study is the largest trial, to date, investigating the consequences of different consent procedures for medical research, patients, and HCPs. We included patients with a wide range of conditions, both malignant and benign, and in both academic and non-academic hospitals. We employed both questionnaires and interviews which, in some cases, yielded different results.

In a previous smaller study of cancer patients, we also investigated the consequences of the same procedures for medical research [9]. In line with that study, the current study showed that patients felt well-informed in both the informed consent and opt-out plus procedure. An important difference in findings is that the smaller study showed a preference for opt-out plus in the majority of respondents, whereas a majority of respondents in the current study indicated a preference for informed consent in the questionnaire. This difference may be due to differences in study samples, although we did not find that type of condition (malignant or benign) was significantly associated with preferences. We speculate that these conflicting findings may result from subtle differences in the way questions were posed in the questionnaires.

In line with previous studies, our study showed biases when informed consent is required [11–15]. Importantly, although the observed biases were statistically significant in our study, this does not imply that the biases we found will be found in all patient groups. Kho et al. [15] showed that, while biases are commonly found when informed consent is required, the direction of the effect differs between studies. Controlling for consent bias to mitigate the effect of informed consent is thus not possible.

Informed consent may negatively affect medical research mostly in health care systems in which consent to treatment is presumed and not explicitly asked, as is the case in the Netherlands. Such health care systems lack the organisation to routinely collect consent forms, and its patients are not used to returning such forms. Interestingly, the consent percentages we found in the informed consent arm of our trial were in the lower regions of percentages found in other studies [7–9].

### Future research

Although our study addressed many important questions, it also raised new ones. Most importantly, we believe that future research should investigate whether variations of the consent procedures could be implemented to maintain their advantages and improve on their shortcomings. An example could be an opt-out plus procedure in which the hospital marks patients’ preferences electronically during registration, thereby reducing costly time of medical personnel.

## Conclusion and policy implications

We conclude that medical research and medical staff are best served by either the opt-out or the opt-out plus procedure. Patients are best served by either the opt-out plus or informed consent procedure; they are well-informed of residual tissue storage and use in both procedures, and importantly, they also feel well-informed. It is, however, not perfectly clear which consent procedure, informed consent or opt-out plus, the majority of patients prefer. Of interest, we found that preferences for consent procedures are not static and can be influenced by new information and previously experienced consent procedures.

Residual tissue use is a complex, multi-faceted issue. In order to fully understand the consequences of specific consent procedures, we must take the effects on all stakeholders into account. Based on our current and previous findings [9], it seems essential that sufficient information is provided to patients, rather than the explicit signature for consent. Taking all of the above into account, we feel that the opt-out plus procedure may be the most optimal in serving the needs of health care today and in the future. Importantly it will allow use of unbiased tissue for research. Hence it provides a good balance between future medical research, patients’ wish to be informed and right to consent, and practicalities in clinical practice.

Ethical approval: The study was approved by the medical ethics committees of all participating centres (The Netherlands Cancer Institute (study ID: P11TIS), VU University Medical Center, Kennemer Gasthuis, Spaarne Hospital, St. Antonius Hospital, and Rode Kruis Hospital). All participants provided written informed consent for the use of their questionnaire data and interview data. A waiver of consent was provided for the randomisation and intervention.

## Supporting Information

S1 CONSORT Checklist (DOC)

S1 Methods (DOCX)

S1 Table Characteristics of patients in the intervention study, respondents to the questionnaire and interviewees. (DOCX)

S2 Table Characteristics of all patients, respondents, and interviewees in the three study arms. (DOCX)

S3 Table Return of consent forms in different subgroups in the informed consent arm (N = 440). (DOCX)

S4 Table Influence of trial arm and interview status on patient’s preferences for a consent procedure*. (DOCX)

S5 Table Topics discussed in questionnaires and during interviews. (DOCX)

S1 Protocol (PDF)

S1 Minimal Data Set Patient data. (SAV)

S2 Minimal Data Set HCP data. (SAV)
