# Supplementary material for: A Randomised Controlled Trial of Consent Procedures for the Use of Residual Tissues for Medical Research: Preferences of and Implications for Patients, Research and Clinical Practice
Source: PLoS One. 2016 Mar 30;11(3):e0152509. doi: 10.1371/journal.pone.0152509 (PMC4814081; doi:10.1371/journal.pone.0152509)
Supplement: S1 Methods — (DOCX) [file pone.0152509.s002.docx]

**S1 Methods**

A total of 1319 eligible patients were randomized between September 2011 and May 2014. All these patients were sent a questionnaire 6 weeks after randomization.

**Questionnaires**

Both the questionnaire and interview contained, among others, questions regarding patients’ experiences with the procedure and their preferences. The paper questionnaire consisted of questions regarding patients’ knowledge about residual tissue use, whether and how they were informed and asked for consent, whether and how patients prefer to be informed and asked for consent, the acceptability of different types of research, agreement with statements about residual tissue, incidental findings, trust in scientific research, diagnosis and tissue removal procedure (e.g. excision, biopsy), demographics, and psychosocial questionnaires (subscales of the Dutch patient satisfaction questionnaire (PSQ-18)[^1^](#_ENREF_1), the Dutch threatening medical situations inventory (TMSI),[^2^](#_ENREF_2) and subscales of the SF-12.[^3^](#_ENREF_3) Questions are summarized in the table below.

**Table: Topics discussed in questionnaires and during interviews**

| **Topic or validated questionnaire** | **Questionnaires** | **Interview** | **Medical staff questionnaire** |
| --- | --- | --- | --- |
| Knowledge questions/statements | X |  |  |
| Written and verbal information about residual tissue use | X | X | X |
| Decisions about residual tissue | X | X |  |
| Consent procedures (experiences and preferences) | X | X | X |
| Acceptability of different types of research (e.g. ‘commercial research’ | X | X |  |
| Ownership feelings | X | X |  |
| Statements related to residual tissue use | X |  |  |
| Return of results | X | X |  |
| Trust | X |  |  |
| Subscale ‘physical functioning’ of the SF-12 | X |  |  |
| Subscale ‘mental health’ of the SF-12 | X |  |  |
| Patient Satisfaction Questionnaire (PSQ-18) | X |  |  |
| Threatening Medical Situations Inventory | X |  |  |
| The patient’s disease or symptoms | X |  |  |
| Previous experiences with tissue removal/donation | X |  |  |

We used two measures for educational level. The first was measured as the percentage of low, intermediate, and highly educated persons living in the patient’s zip code area, a proxy for one’s own education level. We used this measure when we conducted analyses on intervention data. The second measure was the actual educational level as indicated by the respondent in the questionnaire. We used this measure when conducting analyses on questionnaire data. Respondents who indicated they finished primary school, lower vocational training, or lower general training were considered to have a low educational level; respondents with intermediate vocational or intermediate of higher general education to have an intermediate educational level; and respondents who completed higher vocational or university training to have a high educational level.

**Semi-structured telephone interview**

The patient questionnaire contained a form on which participants could indicate whether or not they were willing to participate in a telephone interview. Interviews were held approximately one week after the receipt of the questionnaire. Initially, our plan was to interview 25% of the patients who gave consent for the telephone interview. The first 17 months of the study, we interviewed all consenting. From March 2013 to July 2013, we interviewed 25% of those who gave consent for the interview. After a while it appeared that the absolute number of interviews would turn out too low if we would continue to ask 25% of consenters for an interview. In addition, we noticed an underrepresentation of patients in certain groups of certain hospitals. Therefore, from July 2013 onwards, we decided to interview all consenting patients in underrepresented groups and hospitals until all subgroups were represented in our sample.

The semi-structured telephone interviews, held by AB and SR, and transcribed by AB, consisted of questions regarding patients’ knowledge of residual tissue use, opinions of the best way to inform patients and to ask their consent, acceptability of different types of research, feelings regarding tissue ownership, and incidental findings (see table). Patients’ statements during the interviews (e.g. whether the interviewee felt patients should be informed about residual tissue use) were independently quantified by AB and SR. Dissimilarities were discussed to reach consensus.

The indicated consent procedure preference in the interview was determined by combining the interviewee’s statements regarding whether or not patients should receive information about residual tissue use, and which consent modality (opt-out vs. informed consent) the interviewee preferred to be used in the hospital (see also table 4). We coded interviewees preferring opt-out with information as having a preference for opt-out plus, interviewees preferring opt-out without information as having a preference for opt-out, and interviewees preferring an explicit consent modality with information as having a preference for informed consent.

**HCP questionnaires**

HCPs received a paper questionnaire after they finished including patients to the study. The questionnaire contained questions regarding experiences with the three consent procedures, as well as their preference for either of them.

The time spent informing patients and answering questions in the informed consent arm and opt-out plus arm was calculated as the difference in time spent on the informed consent and opt-out plus procedures with the time spent on the opt-out procedure (see also table 3). We used the average time when the respondent gave a range.

**Analysis**

We considered patient satisfaction (measured as informedness), patient preferences for a consent procedure, percentage of patients aware of residual tissue storage, consent rates (tissue availability and bias), HCP satisfaction, and HCP preferences for a consent procedure as primary outcomes. Secondary outcomes were the percentage of patients indicating that their residual tissue may be used for scientific research, patients’ knowledge about residual tissue use, interference of a consent procedure with giving patients clinical information, and the time physicians spent on informing patients and answering their questions in the three procedures.

1. Marshall GN, Hays RD. The patient satisfaction questionnaire short-form (PSQ- 18): RAND Corporation, 1994.

2. Van Zuuren F, De Groot K, Mulder N, Muris P. Coping with medical threat: An evaluation of the threatening medical situations inventory (TMSI). *Personality and Individual Differences* 1996; **21**: 21-31.

3. Ware J, Kosinski M, Keller SD. A 12-Item Short-Form Health Survey: construction of scales and preliminary tests of reliability and validity. *Med Care* 1996; **34**: 220-33.
