# Supplementary material for: A Randomised Controlled Trial of Consent Procedures for the Use of Residual Tissues for Medical Research: Preferences of and Implications for Patients, Research and Clinical Practice
Source: PLoS One. 2016 Mar 30;11(3):e0152509. doi: 10.1371/journal.pone.0152509 (PMC4814081; doi:10.1371/journal.pone.0152509)
Supplement: S1 Table — (DOCX) [file pone.0152509.s003.docx]

**S1 Table: Characteristics of patients in the intervention study, respondents to the questionnaire and interviewees**

|  | **All patients (N=1319)  %** | **Intervention only (N=646) %** | **Respondents (no interview) (N=527) %** | **Interviewees (N=146) %** | **P-value* difference between the three groups** | **P-value* intervention only vs. respondents** | **P-value* respondents vs. interviewees** |
| --- | --- | --- | --- | --- | --- | --- | --- |
| **Intervention arm** |  |  |  |  | 0.316 | 0.285 | 0.158 |
| Informed consent | 33 | 32 | 36 | 30 |  |  |  |
| Opt-out plus | 33 | 34 | 30 | 38 |  |  |  |
| Opt-out | 34 | 34 | 34 | 32 |  |  |  |
| **Sex** |  |  |  |  | 0.527 | 0.316 | 0.735 |
| Male | 39 | 37 | 40 | 42 |  |  |  |
| Female | 61 | 63 | 60 | 58 |  |  |  |
| **Educational level (questionnaire data)** |  |  |  |  | - | - | 0.342 |
| Low | 9 | - | 18 | 14 |  |  |  |
| Intermediate | 22 | - | 41 | 48 |  |  |  |
| High | 16 | - | 31 | 33 |  |  |  |
| Missing | 53 | 100 | 10 | 6 |  |  |  |
| **Age (years)** |  |  |  |  | <0.001 | <0.001 | 0.004 |
| 18-40 | 19 | 27 | 14 | 6 |  |  |  |
| 41-60 | 32 | 29 | 36 | 30 |  |  |  |
| 61-80 | 47 | 41 | 49 | 63 |  |  |  |
| Missing | 2 | 4 | 2 | 1 |  |  |  |
| **Hospital** |  |  |  |  | <0.001 | <0.001 | 0.003 |
| Netherlands   Cancer Institute | 35 | 31 | 36 | 53 |  |  |  |
| VU University   medical center | 27 | 34 | 19 | 20 |  |  |  |
| Spaarne hospital | 1 | 2 | 0 | 1 |  |  |  |
| Kennemer hospital | 13 | 13 | 15 | 10 |  |  |  |
| St. Antonius   hospital | 21 | 18 | 26 | 15 |  |  |  |
| Rode Kruis   hospital | 3 | 2 | 3 | 2 |  |  |  |
| **Academic vs non-academic hospital** |  |  |  |  | <0.001 | <0.001 | <0.001 |
| Academic hospital | 62 | 65 | 55 | 73 |  |  |  |
| Non-academic  hospital | 38 | 35 | 45 | 27 |  |  |  |
| **Procedure** |  |  |  |  | 0.003 | 0.440 | 0.008 |
| Excision | 49 | 46 | 49 | 64 |  |  |  |
| Biopsy or puncture | 21 | 22 | 20 | 16 |  |  |  |
| Blood withdrawal | 22 | 25 | 22 | 12 |  |  |  |
| Other | 6 | 6 | 7 | 6 |  |  |  |
| Missing | 2 | 2 | 2 | 2 |  |  |  |
| **Benign or malignant disease** |  |  |  |  | <0.001 | 0.055 | 0.001 |
| Malignant disease | 54 | 49 | 56 | 73 |  |  |  |
| Benign disease | 44 | 50 | 43 | 25 |  |  |  |
| Unknown | 1 | 1 | 1 | 1 |  |  |  |
| **Tissue site** |  |  |  |  | <0.001 | 0.060 | 0.027 |
| Dermatological | 45 | 42 | 48 | 53 |  |  |  |
| Otolaryngological | 9 | 11 | 7 | 8 |  |  |  |
| Gastroenterological | 8 | 7 | 9 | 16 |  |  |  |
| Pulmonal | 4 | 4 | 4 | 2 |  |  |  |
| Haematological | 22 | 25 | 22 | 12 |  |  |  |
| Gynaecological | 12 | 13 | 11 | 10 |  |  |  |

*Differences between groups were tested using chi-square tests. Six Dutch hospitals included patients in the trial: two academic hospitals (the Netherlands Cancer Institute, Amsterdam, and the VU University Medical Centre, Amsterdam); and four non-academic hospitals (the Kennemer Gasthuis, Haarlem, the Spaarne Hospital, Hoofddorp, the St. Antonius Hospital, Nieuwegein and the Rode Kruis Hospital, Beverwijk). Some HCPs preferred to include only a subset of eligible patients due to time constraints. For example, in the Netherlands Cancer Institute, patients with skin excisions but not skin biopsies were included.
