# Supplementary material for: A Randomised Controlled Trial of Consent Procedures for the Use of Residual Tissues for Medical Research: Preferences of and Implications for Patients, Research and Clinical Practice
Source: PLoS One. 2016 Mar 30;11(3):e0152509. doi: 10.1371/journal.pone.0152509 (PMC4814081; doi:10.1371/journal.pone.0152509)
Supplement: S2 Table — (DOCX) [file pone.0152509.s004.docx]

**S2 Table: Characteristics of all patients, respondents, and interviewees in the three study arms**

|  | **All patients (N=1319)** | | | | **Respondents (N=673)** | | | | **Interviewees (N=146)** | | | |
| --- | --- | --- | --- | --- | --- | --- | --- | --- | --- | --- | --- | --- |
|  | Inf. consent arm | Opt-out plus arm | Opt-out arm |  | **Inf. consent arm** | **Opt-out plus arm** | **Opt-out arm** |  | **Inf. consent arm** | **Opt-out plus arm** | **Opt-out arm** |  |
|  | **N=440** | **N=434** | **N=445** |  | **N=233** | **N=215** | **N=225** |  | **N=44** | **N=56** | **N=46** |  |
|  | **%** | **%** | **%** | **P-value*** | **%** | **%** | **%** | **P-value*** | **%** | **%** | **%** | **P-value*** |
| Questionnaire returned | 53 | 50 | 51 | 0.584 | 100 | 100 | 100 | - | 100 | 100 | 100 | - |
| Interviewed | 10 | 13 | 10 | 0.327 | 19 | 26 | 20 | 0.158 | 100 | 100 | 100 | - |
| **Sex** |  |  |  | 0.696 |  |  |  | 0.369 |  |  |  | 0.463 |
| Male | 38 | 39 | 38 |  | 44 | 39 | 38 |  | 48 | 36 | 44 |  |
| Female | 62 | 60 | 62 |  | 56 | 61 | 62 |  | 52 | 64 | 57 |  |
| **Age (years)** |  |  |  | 0.988 |  |  |  | 0.796 |  |  |  | 0.917 |
| 18-40 | 19 | 19 | 19 |  | 12 | 12 | 12 |  | 5 | 7 | 7 |  |
| 41-60 | 32 | 32 | 32 |  | 38 | 33 | 34 |  | 34 | 30 | 26 |  |
| 61-80 | 48 | 46 | 46 |  | 49 | 54 | 53 |  | 61 | 61 | 67 |  |
| Missing | 1 | 4 | 4 |  | 0 | 2 | 1 |  | 0 | 2 | 0 |  |
| **Educational level (questionnaire data)** |  |  |  | 0.327 |  |  |  | 0.327 |  |  |  | 0.602 |
| Low | 8 | 8 | 9 |  | 16 | 16 | 19 |  | 18 | 14 | 9 |  |
| Intermediate | 22 | 24 | 20 |  | 41 | 48 | 40 |  | 43 | 52 | 48 |  |
| High | 18 | 13 | 17 |  | 33 | 27 | 34 |  | 32 | 29 | 39 |  |
| Missing | 52 | 55 | 53 |  | 10 | 9 | 8 |  | 7 | 5 | 4 |  |
| **Hospital** |  |  |  | 1.000 |  |  |  | 0.842 |  |  |  | 0.185 |
| Netherlands  Cancer Institute | 36 | 36 | 35 |  | 42 | 40 | 36 |  | 57 | 54 | 48 |  |
| VU University   medical center | 26 | 27 | 27 |  | 19 | 20 | 20 |  | 16 | 16 | 28 |  |
| Spaarne hospital | 2 | 1 | 1 |  | 0 | 1 | 0 |  | 0 | 2 | 0 |  |
| Kennemer   hospital | 13 | 13 | 13 |  | 13 | 13 | 16 |  | 18 | 5 | 7 |  |
| St. Antonius   hospital | 21 | 21 | 21 |  | 23 | 23 | 25 |  | 7 | 20 | 17 |  |
| Rode Kruis   hospital | 3 | 3 | 3 |  | 4 | 3 | 2 |  | 2 | 4 | 0 |  |
| **Academic vs non-academic hospital** |  |  |  | 0.878 |  |  |  | 0.670 |  |  |  | 0.768 |
| Academic   hospital | 61 | 62 | 62 |  | 60 | 60 | 56 |  | 73 | 70 | 76 |  |
| Non-academic   hospital | 39 | 38 | 38 |  | 40 | 40 | 44 |  | 27 | 30 | 24 |  |
| **Procedure** |  |  |  | 0.955 |  |  |  | 0.412 |  |  |  | 0.181 |
| Excision | 49 | 49 | 50 |  | 55 | 49 | 53 |  | 73 | 63 | 59 |  |
| Biopsy or   punction | 20 | 21 | 20 |  | 16 | 25 | 17 |  | 14 | 14 | 20 |  |
| Blood   withdrawal | 22 | 23 | 22 |  | 20 | 21 | 19 |  | 2 | 20 | 13 |  |
| Other | 7 | 7 | 5 |  | 7 | 6 | 7 |  | 9 | 4 | 4 |  |
| Missing | 2 | 1 | 3 |  | 1 | 1 | 4 |  | 2 | 0 | 4 |  |
| **Benign or malignant disease** |  |  |  | 0.753 |  |  |  | 0.974 |  |  |  | 0.878 |
| Malignant   disease | 54 | 55 | 55 |  | 60 | 61 | 58 |  | 77 | 71 | 72 |  |
| Benign disease | 45 | 44 | 44 |  | 39 | 38 | 40 |  | 23 | 27 | 26 |  |
| Missing/   unknown | 1 | 2 | 1 |  | 1 | 1 | 1 |  | 0 | 2 | 2 |  |
| **Tissue site** |  |  |  | 1.000 |  |  |  | 0.967 |  |  |  | 0.232 |
| Dermatological | 45 | 45 | 46 |  | 47 | 47 | 52 |  | 57 | 48 | 54 |  |
| Otolaryngolo-   gical | 8 | 9 | 9 |  | 7 | 7 | 7 |  | 11 | 4 | 9 |  |
| Gastroenterolo-  gical | 9 | 8 | 8 |  | 10 | 11 | 9 |  | 18 | 14 | 15 |  |
| Pulmonal | 4 | 4 | 4 |  | 3 | 4 | 3 |  | 0 | 2 | 4 |  |
| Haematological | 22 | 22 | 22 |  | 20 | 21 | 19 |  | 2 | 20 | 13 |  |
| Gynaecological | 12 | 12 | 11 |  | 13 | 9 | 10 |  | 11 | 13 | 4 |  |

* Differences tested using chi-square tests between the three study arms. Inf. consent=informed consent
