# Supplementary material for: A Randomised Controlled Trial of Consent Procedures for the Use of Residual Tissues for Medical Research: Preferences of and Implications for Patients, Research and Clinical Practice
Source: PLoS One. 2016 Mar 30;11(3):e0152509. doi: 10.1371/journal.pone.0152509 (PMC4814081; doi:10.1371/journal.pone.0152509)
Supplement: S3 Table — (DOCX) [file pone.0152509.s005.docx]

**S3 Table: return of consent forms in different subgroups in the informed consent arm (N=440)**

|  | **Return of consent form** |
| --- | --- |
|  | **%** |
| **Sex** |  |
| Male | 69 |
| Female | 56 |
| **Educational level (questionnaire data)*** |  |
| Low | 86 |
| Intermediate | 68 |
| High | 71 |
| **Age (years)** |  |
| 18-40 | 45 |
| 41-60 | 60 |
| 61-80 | 68 |
| **Academic vs non-academic hospital** |  |
| Academic hospital | 54 |
| Non-academic hospital | 72 |
| **Procedure** |  |
| Excision | 62 |
| Biopsy or puncture | 63 |
| Blood withdrawal | 56 |
| Other | 65 |
| **Benign or malignant disease** |  |
| Malignant disease | 61 |
| Benign disease | 61 |
| Unknown | 67 |
| **Tissue site** |  |
| Dermatological | 70 |
| Otolaryngological | 53 |
| Gastroenterological | 51 |
| Pulmonal | 44 |
| Haematological | 57 |
| Gynaecological | 55 |

*Educational data from the zip code method is continuous and therefore not shown here
