# Supplementary material for: A Randomised Controlled Trial of Consent Procedures for the Use of Residual Tissues for Medical Research: Preferences of and Implications for Patients, Research and Clinical Practice
Source: PLoS One. 2016 Mar 30;11(3):e0152509. doi: 10.1371/journal.pone.0152509 (PMC4814081; doi:10.1371/journal.pone.0152509)
Supplement: S4 Table — (DOCX) [file pone.0152509.s006.docx]

**S4 Table: Influence of trial arm and interview status on patient’s preferences for a consent procedure***

|  | **OR (95% CI)** | **P-value** |
| --- | --- | --- |
| **Consent procedure preference by trial arm** | | |
| *Preference for informed consent vs. opt-out (reference)* | | |
| Informed consent arm | Reference |  |
| Opt-out arm | 0.667 (0.346-1.283) | 0.225 |
| Opt-out plus arm | 0.407 (0.215-0.768) | 0.006 |
| *Preference for informed consent vs. opt-out plus (reference)* | | |
| Informed consent arm | Reference |  |
| Opt-out arm | 0.602 (0.388-0.935) | 0.024 |
| Opt-out plus arm | 0.369 (0.238-0.572) | <0.0001 |
| *Preference for opt-out plus vs. opt-out (reference)* | | |
| Informed consent arm | Reference |  |
| Opt-out arm | 1.107 (0.545-2.250) | 0.779 |
| Opt-out plus arm | 1.101 (0.560-2.164) | 0.781 |
| **Consent procedure preference by interview status** | | |
| *Preference for informed consent vs. opt-out (reference)* | | |
| Non-interviewees | Reference |  |
| Interviewees | 0.578 (0.322-1.035) | 0.065 |
| *Preference for informed consent vs. opt-out plus (reference)* | | |
| Non-interviewees | Reference |  |
| Interviewees | 0.586 (0.389-0.883) | 0.011 |
| *Preference for opt-out plus vs. opt-out (reference)* |  |  |
| Non-interviewee | Reference |  |
| Interviewees | 0.985 (0.539-1.798) | 0.960 |

*Results based on six logistic regression analyses. Respondents with no preference or who did not know which procedure to prefer were omitted from the analysis; OR=Odds Ratio; CI=Confidence Interval
