# Supplementary material for: A Randomised Controlled Trial of Consent Procedures for the Use of Residual Tissues for Medical Research: Preferences of and Implications for Patients, Research and Clinical Practice
Source: PLoS One. 2016 Mar 30;11(3):e0152509. doi: 10.1371/journal.pone.0152509 (PMC4814081; doi:10.1371/journal.pone.0152509)
Supplement: S5 Table — (DOCX) [file pone.0152509.s007.docx]

**S5 Table: Topics discussed in questionnaires and during interviews**

| **Topic or validated questionnaire** | **Questionnaires** | **Interview** | **Physician questionnaire** |
| --- | --- | --- | --- |
| Knowledge questions/statements | X |  |  |
| Written and verbal information about residual tissue use | X | X | X |
| Decisions about residual tissue | X | X |  |
| Consent procedures (experiences and preferences) | X | X | X |
| Acceptability of different types of research (e.g. ‘commercial research’ | X | X |  |
| Ownership feelings | X | X |  |
| Statements related to residual tissue use | X |  |  |
| Return of results | X | X |  |
| Trust | X |  |  |
| Subscale ‘physical functioning’ of the SF-12 | X |  |  |
| Subscale ‘mental health’ of the SF-12 | X |  |  |
| Patient Satisfaction Questionnaire (PSQ-18) | X |  |  |
| Threatening Medical Situations Inventory | X |  |  |
| The patient’s disease or symptoms | X |  |  |
| Previous experiences with tissue removal/donation | X |  |  |
