# Supplementary material for: A Randomised Controlled Trial of Consent Procedures for the Use of Residual Tissues for Medical Research: Preferences of and Implications for Patients, Research and Clinical Practice
Source: PLoS One. 2016 Mar 30;11(3):e0152509. doi: 10.1371/journal.pone.0152509 (PMC4814081; doi:10.1371/journal.pone.0152509)
Supplement: S1 Protocol — (PDF) [file pone.0152509.s008.pdf]

NKI-AVL

Protocol

The Tissue Issue: towards a uniform consent procedure for research with excised (cancer) tissue

KWF project NKI 2009 - 4535

Date: May 5th 2011

Principal investigators:

Dr. ir. M.K. Schmidt (NKI-AVL)
Prof. dr. ir. F.E. van Leeuwen (NKI-AVL)
Prof. dr. N.A. Aaronson (NKI-AVL)
Dr. E. Vermeulen (VUmc)

Contact and coordinator:

Susanne Rebers
Nederlands Kanker Instituut - Antoni van Leeuwenhoek ziekenhuis
Psychosociaal Onderzoek en Epidemiologie (PSOE)
Visiting address: Plesmanlaan 121, 1066 CX Amsterdam
Postal address: Postbus 90203, 1006 BE, Amsterdam
Telephone: 020- 512 2485
Fax: 020- 512 2322
E-mail: s.rebers@nki.nl

Summary KWF project “The Tissue Issue” 4
Background 4
Study objectives 4
Methods 4
Results/relevance 5
Introduction 6
Use of (residual) human materials in research 6
Regulation of use of residual human materials in research 6
Preliminary results 7
Study objectives 8
Hypotheses 8
Psychosocial measures 9
Outcomes 9
Patients and methods 10
Study sample 10
Study design including study arms 10
The intervention: 3 consent procedures 11
Standardized texts (see attachments O and P for Dutch versions) 12
Questionnaires and interview 12
Data collection: first questionnaire (see attachment B) 13
Data collection: interview 14
Data collection: second questionnaire (see attachment C) 14
Data collection: questionnaires for the medical staff 14
Sample size, statistical power 14
Basic plan of analysis 15
Privacy 16
Publication policy 16
Burden for the patients / respondents 16
List of attachments 17
Figures 18
References 20

We acknowledge the following people who commented on (parts of) this protocol:

Tessa van der Valk (VSOP)
Pauline Evers (NFK)
Members of the ‘commissie patiëntenparticipatie in wetenschappelijk onderzoek’ (NFK)
Corrette Ploem (AMC)
Suzanne Gerretsen (NKI-AVL)
Heleen Hauer (NKI-AVL)
Frits Lekkerkerker (chairman commissie patiëntenparticipatie in wetenschappelijk onderzoek (NFK) and chairman NVMETC)

Summary KWF project "The Tissue Issue"

Background

Human samples obtained during standard clinical care, such as tissue from surgery or blood, are being increasingly collected, banked and used in basic, translational and clinical research. This tissue is referred to as *residual* tissue, as compared to (extra) tissue *specifically* collected for scientific research. There is currently no standard procedure in the Netherlands that prescribes whether and how patients should be asked consent for the use of residual tissue. Empirical data about patients' preferences and wishes regarding information and consent procedures for such use is scarce, and the available research results have not been translated to clinical practice.

Study objectives

This KWF-funded project aims to determine patient preferences for and satisfaction with consent procedures regarding the use of residual tissue for research; to provide insight into the type of information patients need to decide on the use of their material in research; and to determine the feasibility of the timing and content of these procedures in clinical practice. We will compare three consent procedures with respect to several study outcomes: consent rates, and patients' and medical staff's satisfaction with and preferences for these procedures.

Methods

In this multicentre, randomized controlled trial, 1800 patients will be recruited from four hospitals in the Netherlands (expected response rate 75%). Patients will be randomized to three study arms. In the 'informed consent' and 'opt-out plus' arms, patients are informed about residual tissue use both verbally and through a specific leaflet. These leaflets contain a consent form and an opt-out form respectively. In the third arm, the control group, no (extra) information about residual tissue use is offered, and patients may opt-out of the use of their residual tissue verbally or written. Patients to be included will be stratified by hospital and condition. Two academic and two regional hospitals will be included. Inclusion of both categories of hospitals and of patients with malignant and non-malignant diseases will increase the generalizability of the study results.

Six weeks after the intervention, patients will receive a questionnaire addressing satisfaction with and preference for the consent procedures. One quarter of the respondents will be interviewed about these topics through a semi-structured telephone interview. Six months after the intervention, respondents will receive a second questionnaire to examine whether those who made a decision about the use of their residual tissue are still aware of that decision and, if they are, whether they are still satisfied with it.

Clinicians and nurses who will carry out the interventions will receive a pre- and post-experimental questionnaire to examine their opinions about and experiences with the consent procedures.

Results/relevance

Expected result of this study will be an information procedure that is in accordance with patients' wishes and research needs and which can be uniformly applied in all Dutch hospitals. This will contribute to the debate about the improvement of regulations (by law or self-regulation) regarding the use of residual tissue for research.

Introduction

Use of (residual) human materials in research

Human samples obtained during standard clinical care, such as tissue from surgery or blood, are being increasingly collected, banked and used in basic, translational and clinical research.[1] This tissue is referred to as *residual* tissue, as compared to (extra) tissue *specifically* collected for scientific research. Significant medical progress has been made based on studies of tissue samples, especially in cancer research.[e.g. 2,3]

Consent for use of stored tissue for research purposes can be obtained in a variety of ways, and there is on-going discussion about which procedure complies best with patients' wishes while also being feasible in clinical practice.[4] Some argue that implied consent with the opportunity to opt-out is sufficient.[5-8] Under opt-out, information about research with tissue is provided but patients only need to inform their physician or hospital if they do not want their tissue to be used. Others claim that explicit one-time *general* (for all future research) consent should be asked.[9-11]

Data about patients' preferences and wishes regarding information and consent procedures for tissue use are available primarily from trials in which tissue or blood was taken specifically for research purposes. Much less research on these issues has been carried out in hospital-based settings where *residual* tissue or blood obtained during diagnostics or treatment may be used for research. In addition, the research results that have been obtained have not been translated to clinical practice.

Regulation of use of residual human materials in research

Two important laws regulating tissue use in the Netherlands are the Dutch Medical Treatment Act (WGBO) and the Medical Research Involving Human Subject Act (WMO). According to the Dutch Medical Treatment Act,[12] anonymous samples obtained during medical treatment may be used in medical research if the patient has not objected to this secondary use. Active consent for use of such materials is not required and the law does not specify how patients should be informed about secondary use of tissue. The Medical Treatment Act does not regulate the use of coded samples. This is done by the 'Code of Conduct for proper secondary use of human tissue in research' published by the Dutch Federation of Biomedical Scientific Societies, together with the Royal Dutch Medical Association and several patient groups.[13] According to this 'Code of Conduct', the use of *residual* tissue is regulated by an 'opt-out' procedure, as in many other countries. Patients can declare that they do not agree with the use of their tissue on the basis of information which in most hospitals is available in a general leaflet. In the future however, use of residual tissue might be regulated by the Human Tissue Act that the Dutch government has been working on for several years now.

Research with tissue *specifically* collected for research is regulated by the Medical Research Involving Human Subjects Act (WMO). This act states that respondents should always be asked written informed consent in this situation (http://www.ccmo-online.nl/main.asp).

During the formulation of the Medical Treatment Act it was debated whether it would be preferable to establish a procedure based on 'one-time general consent'.[14,15] The report of the Royal Dutch Academy of Sciences (KNAW) on multifactorial diseases concluded that the

opt-out regime is preferable, largely based upon concerns that asking for one time general consent would not be feasible in the clinical setting and that therefore consent would not be obtained for a substantial proportion of patients.[16] Moreover, there is empirical evidence that specific groups of patients would likely decline consent and that the tissues available for research would be biased.[e.g. 17] Another problem related to obtaining consent via the one-time consent procedure is the burden for the hospital staff which may lead to missing forms.[18] The Advisory Council on Health Research (RGO) supported the conclusion of KNAW and advised in 2007 to formulate the new Human Tissue Act based on Federa's Code of Conduct.[19] However, some health lawyers state that patients should be able to give or withhold active consent.[15] As such, proposed changes in Dutch regulation on the use of these samples, i.e. the obligation to obtain (one-time) informed consent from all 'donors' involved, may hamper future research. It has been argued that such an informed consent procedure will not be to the benefit of patients or researchers.[16,19] Moreover, many patients do not believe informed consent is necessary. [20-22]

A disadvantage of the current opt-out procedure, however, is that patients are generally not aware that tissue is stored for a future diagnosis.[23] This is an important deficit when considering the importance of stored tissue for an eventual future diagnosis of the patient or her/his relatives. The fact that patients are not aware of tissue storage also signals the fact that most patients are not aware that they can opt-out (declare that they do not agree to the use of their extracted and stored tissue in medical research). In our exploratory research (see below) we therefore devised and tested a procedure that ensures that patients are aware of storage and research with residual tissue. This procedure, opt-out plus, involves verbal information and a leaflet about tissue research. With this information the patient can consider opting-out. In contrast to informed consent, the opt-out plus procedure does not require much administrative work because there is no need to process consent forms from all patients. Moreover, the opt-out plus procedure may not lead to lower consent rates and bias in the available tissue, as is very likely the case with the informed consent procedure, because this procedure does not depend on an action taken by patients.

Preliminary results

Over the last years our research group has conducted three explorative studies about consent and patient information.[21-23] We examined how patients in the NKI-AVL and VUmc with breast cancer, prostate cancer and colorectal cancer wished to be informed about research with stored tissue, and what kind of consent procedure they preferred. The studies showed that most respondents (96-98%) consented to research with their tissue. This is in line with other research.[8,9,24-28] In the first study, patients were asked whether they preferred informed consent or opt-out.[21] Most patients preferred a consent procedure with active information, but did not consider the signing of a consent form important, especially because they endorsed research and wanted to minimize bureaucratic burden. The respondents in these studies suggested a procedure that was labelled 'opt-out plus': patients receive verbal and written information, a special leaflet about tissue and research, and the option to opt-out.

In the two subsequent studies, the opinion of patients about the three consent procedures - informed consent, opt-out and opt-out plus- was explored by means of written questionnaires and telephone interviews. Further, the influence of these procedures on consent rates and availability of residual tissues for scientific research was studied. The majority of patients

preferred opt-out plus over the two other procedures. Opt-out and opt-out plus resulted in the highest availability of tissue for scientific research.

Previous studies thus indicate that opt-out plus is promising because it places few demands on administrative resources of the medical staff of hospitals while addressing the information needs of patients. These studies were, however, exploratory in nature and included small numbers of selected surgical cancer patients. In order to determine the best way of informing Dutch patients (with various diagnoses) the three consent procedures will be evaluated in this study in more diverse patient populations and different settings. The outcome of the current KWF-funded 4-year study will provide evidence on the patients', clinicians', and researchers' points of view that can be used in the current debate about new regulations regarding residual tissue use. The results may guide the implementation of a nationwide consent procedure and information leaflet for research with residual tissues. It is clearly in the interest of patients, clinicians and researchers to determine the most feasible, effective and acceptable way to gain consent from patients for the use of their residual tissue in medical research.

A more detailed overview of the background of this study and of the results of previous studies in our research group on consent procedures can be found in attachment A.

Study objectives

The main part of this study aims to determine patient preferences for and satisfaction with consent procedures regarding the use of residual tissue for research, to provide insight into the type of information patients need to decide on the use of their material in research, and to determine the optimality of the timing and the content of these procedures in clinical practice. We will compare the three consent procedures, opt-out, opt-out plus and informed consent, with respect to the following primary study outcomes:

1. Patients' satisfaction with and preference for a consent procedure
2. Proportion of patients aware of storage of tissue and research use
3. Consent rates
4. Medical staff's satisfaction with and preference for a consent procedure

Additionally, we will obtain information on (1) the proportion of non-consent due to non-response; (2) the characteristics of non-consenters and non-respondents; and (3) the reasons for providing or withholding consent or (non-)response. We will investigate how patients perceived the information they received about residual tissue use and their knowledge about this topic. Patients' preferences to provide or withhold consent for specific types of research (e.g. genetic, commercial) and feedback regarding unanticipated findings will be investigated as well. Further, we will examine whether consent rates, satisfaction with or preference for a consent procedure differ according to severity of disease, type of clinical procedure (e.g., biopsies or surgical removal of tissue), diagnosis, type of hospital (academic or community) or demographics. Six months after the first questionnaire, we will examine whether patients are still aware of the decision they made and whether they are still satisfied with their choice to provide or withhold consent.

Hypotheses

Hypotheses regarding the primary study outcomes are:

1. Patients' will be significantly more satisfied with the informed consent and opt-out plus consent procedures than with the opt-out consent procedure
2. Most patients will prefer the opt-out plus consent procedure, the lowest proportion of patients will prefer the opt-out consent procedure.
3. Significantly more patients will be aware of storage of tissue and research use in the informed consent and opt-out plus procedures than in the opt-out procedures.
4. Consent rates will be highest in opt-out and opt-out plus.
5. Members of the medical staff will be most satisfied with and prefer the opt-out and opt-out plus procedures.

Psychosocial measures

Although some research has been conducted on the relation between specific psychosocial measures and patient preferences regarding information about their disease[e.g. 29] or regarding medical decision making,[e.g. 30,31] to the best of our knowledge, no research has been conducted on the relation between such measures, such as patient satisfaction or coping style, and preferences for consent procedures for the use of residual tissues. Therefore, we add several psychosocial measures to our questionnaire. We consider this part of our study exploratory in nature.

This part of the study will investigate whether physical functioning, mental health, satisfaction with medical care, coping style and one's psychological reaction to stressful events influence a patient's preferences for and satisfaction with consent procedures. Moreover, we will investigate whether these variables influence consent rates.

Outcomes

The primary study outcomes will be used to determine which consent procedure serves the needs of patients, clinicians and scientific research best. If one of the three consent procedures serves the needs of all three of these parties best, we will conclude that this consent procedure is the best candidate to become the standard procedure in the Netherlands that prescribes whether and how patients should be asked consent for the use of residual tissue. If the needs of these three parties are best served by different consent procedures, or if it is unclear which consent procedure serves the needs of one or more of the parties best, we will organize an invitational conference in which we invite different stakeholders, such as clinicians, patient organizations and researchers, to discuss how to translate our research findings into clinical practice.

Which consent procedure serves a party's needs best will be decided along the following lines:

- For patients: satisfaction with the received information in the 'best procedure' should be significantly higher than in the consent procedure with the lowest satisfaction rates. Further, the consent procedure should have the highest preference rates among respondents, with a minimum of a 10% difference with the consent procedure with the lowest preference rate. Additionally, the proportion of patients aware of storage of tissue and research use should be at least as high as in the other two groups.

- For clinicians: satisfaction with the received information in the ‘best procedure’ should be significantly higher than in the consent procedure with the lowest satisfaction rates. Further, the consent procedure should have the highest preference rates among participating clinicians and nurse practitioners, with a minimum of a 10% difference with the consent procedure with the lowest preference rate. Additionally, more than 75% of the responding medical staff should indicate that the consent procedure ‘never’ or ‘seldom’ interferes with giving clinical information.
- For research: consent rates for the procedure for which patients and/or clinicians have the highest preference should be at least as high as consent rates in the two other procedures.

Patients and methods

Study sample

The study sample will be composed of 1800 patients, evenly distributed over the three study arms. Sample size is based on an expected response rate (for the questionnaire) of 75%. We will include patients who undergo surgery or biopsy (1600 patients) or blood drawing (200 patients). The former category of patients will contain patients who have a skin condition, a colorectal condition or a condition in the head/neck area. We include patients with malignant and non-malignant conditions of the same tissues or organs with a ratio of about 3:2, dependent on availability in each hospital. The absolute number of patients with specific conditions will also depend on the availability of these patients in the four hospitals. In the VUmc we will not include patients with (malignant and benign) colorectal conditions and malignant head/neck conditions, because almost all of them will also be asked to participate in a biobank (String of Pearls Initiative), which might confuse patients about what they are asked to give consent for. In the NKI-AVL, only patients with malignant conditions will be included. Figure 1 gives an overview of the number of patients in each of the groups in the four hospitals.

We will include patients between the ages of 18 and 80 years. There are two reasons for the upper age limit. First, we have learned from experience that patients over 80 are much less likely to be able or willing to complete questionnaires. Second, and more importantly, the two community hospitals in our study treat more patients over 80 than the two academic hospitals. Including these patients would mean that it would be difficult to determine whether any differences observed between patients from these different types of hospitals should be attributed to age differences or other causes.

Patients should be able to speak and read Dutch and have a sufficient mental capacity to complete the questionnaires by themselves. Patients will be included in the study by their treating physician or nurse practitioner.

Study design including study arms

In this multicentre randomized controlled trial, 1800 patients will be recruited from academic and community hospitals during an 18 month period (Figure 2). The consent procedures will be offered a short time before or after the tissue or blood is removed, dependent on what is the best timing according to the clinician or nurse practitioner. Patients to be included are

distributed over four hospitals: two academic hospitals and two regional hospitals: the NKI-AVL, the Vrije Universiteit Medical Center (VUmc), the Spaarne hospital in Hoofddorp and the Kennemer hospital in Haarlem. Inclusion of academic and community hospitals will increase the external validity (i.e. generalizability) of the study results.

Patients will be randomized, stratified by hospital and diagnosis, to opt-out plus, informed consent or opt-out (control group) in collaboration with Harm van Tinteren (statistician NKI-AVL). Clininians and nurse practitioners who will carry out the intervention will receive envelopes that each contain the paperwork (checklist, brochure in opt-out plus and informed consent arms and return envelopes) for one patient. The envelopes will be put in order of the randomization. Clinicians and nurse practitioners will write down his or her name, the patient's name, patient number, diagnosis, date and method of tissue removal on the checklist. A random selection of diagnoses on the checklists will be compared with the diagnoses in the medical files to check their validity. If the validity is found to be poor, all diagnoses will be checked.

In our previous intervention study (see preliminary results) we asked patients informed consent before the start of the intervention. However, patients felt that it was confusing to be asked informed consent for a study in which they were asked to provide informed consent. To prevent this kind of confusion in the current study, we will not ask consent for the randomization and the intervention. We believe that not asking consent for the randomization is suitable to our study because the informed consent procedure bears no potential harm to the participants, the intervention is of importance for future clinical research, the intervention includes no invasive procedure for the participants, and at least the standard care is offered to participants in the control group.

After the intervention, we will include an informed consent form in the first questionnaire. With this form, we ask consent for the first and second questionnaire (see below). At the end of the questionnaire permission will be asked for a telephone interview.

The intervention: 3 consent procedures

Patients who receive the opt-out plus procedure (the patient is actively informed about the option to opt-out from research with residual tissue) and the informed consent procedure (the patient is asked for broad written consent for research with residual tissue) will receive specific information from their treating physician or a nurse practitioner by using a standardized text and a special information leaflet which is more extensive than the information leaflets currently available in the hospitals. In principle, this information will be given during a planned visit at the clinic. When this is impossible, the intervention might be conducted through telephone.

The consent procedures:
1. Opt-out plus. In this procedure patients will receive verbal and written information, and a specific leaflet (attachment F) on the use of residual tissue in research including an opt-out form. Patients can opt-out by returning this form using a stamped envelope.

2. Informed consent. In this procedure patients will receive verbal and written information and a specific leaflet (attachment G) on the use of residual tissue in research with a consent form to provide explicit written consent. Patients are asked to return the consent form using the

stamped envelope, indicating whether or not they consent to the use of their residual tissue in future research.

3. Opt-out (control). This group of patients will receive usual care. They may read information about the use of residual tissue in research in the (general) information leaflet of the hospital.

Patients in the two intervention arms will be informed by the medical staff using a standardized text (see below), which takes approximately one minute.

Standardized texts (see attachments O and P for Dutch versions)

1. Opt-out plus: “Shortly <<tissue type>> will be removed. This tissue will be examined and stored in case it is necessary for your treatment that it is examined again. This tissue may also be used in scientific research. We want to ask you whether your stored tissue may be used for scientific research. It is important for the hospital that tissue is available for scientific research, because research on new methods of treatment and diagnosis often uses residual tissue. This brochure contains more information about residual tissue. Could you read the brochure? If your residual tissue can be used for scientific research, you do not have to do anything. If you do not want your residual tissue to be used for scientific research, you can fill in the form attached to the brochure and return it to us in the stamped envelope.”

2. Informed consent: “Shortly <<tissue type>> will be removed. This tissue will be examined and stored in case it is necessary for your treatment that it is examined again. This tissue may also be used in scientific research. We want to ask you whether your stored tissue may be used for scientific research. It is important for the hospital that tissue is available for scientific research, because research on new methods of treatment and diagnosis often uses residual tissue. This brochure contains more information about residual tissue. Could you read the brochure, indicate whether or not your residual tissue can be used for scientific research, and return the form to us in the stamped envelope?”

Questionnaires and interview

Data on patient characteristics, medical history and experiences with research and genetic testing will be obtained through a questionnaire, while clinical data, including the diagnosis, will be obtained from the medical records of patients. All recruited patients will be asked to complete a questionnaire (see below) six weeks after the intervention or six weeks after surgery for the control group. The questionnaire will be mailed to their home address, which will be retrieved from the patients’ medical files. For non-respondents a written reminder will be sent after one month and a second reminder will be given by telephone (including a structured non-response item list).

In 25% of the respondents, including all participants who did not consent to research with their residual tissues, a structured telephone interview (see below) will be conducted (provided respondents gave permission to be interviewed) to obtain supplementary, qualitative data about the experience with the consent procedure. These respondents will be equally distributed over hospitals, conditions and intervention arms, besides the respondents who did not consent to research with their tissues. Mixed methods research is especially suited for exploring complex situations and allows for a representation of patient experience and preferences both in quantitative (social facts paradigm) and qualitative (social

construction paradigm) data. The method is used because consent procedures (specifically the difference between opting-out and providing written consent) are complex matters which may be evaluated by patients in a different way according to situation but especially according to the type of research for which consent is solicited (see also preliminary results). Further, all respondents will be sent a second questionnaire (see below) six months after their decision (i.e. consent or not, or opt-out or not) to assess whether they are still satisfied with the decision they made earlier.

Data collection: first questionnaire (see attachment B)

The first questionnaire consists of several parts. All questions have been developed for this study, based on the questionnaire of our previous intervention study, unless otherwise indicated.

- Questions to measure patients' knowledge about residual tissue use. These questions have been developed for this study.
- Questions about how patients perceived the information about residual tissue use (both brochures and verbal information) they received, and how they would have liked to be informed.
- Questions to assess whether patients were aware of the fact that they could have had an influence on residual tissue use and if so, which decision they made.
- Questions to assess patients' satisfaction with the consent procedure and their preferences for a consent procedure.
- Questions about what types of research patients want to give consent for (commercial research, different types of diseases, research in other hospitals), and how often they want to give consent (once or for each new protocol).
- Questions to assess feelings of ownership of the tissue, trust in research or the hospital, and how important they feel scientific research is. Some of these questions were developed by the Rathenau Institute[32].
- Questions to assess whether patients want feedback on unexpected findings in their residual tissue and whether not receiving feedback would influence their decision about residual tissue use.
- Questions about previous experiences with scientific research and tissue removal. Some of these questions were developed by the Rathenau Institute[32].
- Questions about demographics.
- Two subscales of the SF-12[33] to assess physical functioning and mental health.
- The PSQ-18, or Patient Satisfaction Questionnaire 18 will be used to assess patient satisfaction with medical care. The questionnaire has been developed based on a larger questionnaire, the PSQ-III. The original, English version of the PSQ-18 has been validated and demonstrated to be reliable (http://www.rand.org/content/dam/rand/pubs/papers/2006/P7865.pdf). Further, the Dutch version of the PSQ-III (Patienten Tevredenheidsvragenlijst) has been validated and demonstrated to be reliable [37]. In this study, we will use three subscales of this scale, namely general satisfaction, interpersonal manner, and communication. Each subscale consists of two items.
- The Threatening Medical Situations Inventory (TMSI) to assess two ways in which people can cope with medical threat: monitoring, or cognitive confrontation, and blunting, or cognitive avoidance. Generally patients with a monitoring style tend to cope with (medical) stress by seeking information, while those with a blunting style cope by avoiding information. The questionnaire consists of four scenarios of medical

threat situations, followed by three monitoring and three blunting items that need to be answered on a five-point scale. The TMSI has been validated and demonstrated to be reliable in several groups of people, both patients and non-patients [38,39].

Data collection: interview

The semi-structured telephone interview schedule broadly covers the same topics as the questionnaire, but in more depth.

Data collection: second questionnaire (see attachment C)

The second questionnaire consists of:

- Questions to assess whether patients are aware of the fact that they could have had an influence on residual tissue use and if so, whether they still remember which decision they made, and whether they are still satisfied with this decision.
- Questions to assess how it would be for patients to be asked consent for a research protocol or to receive information on an unexpected finding some time after surgery.

Data collection: questionnaires for the medical staff

The opinions of the medical staff (treating physicians and nurse practitioners) about the different consent procedures and the associated workload will be evaluated by a brief, structured questionnaire administered before and after the interventions.

The pre-intervention questionnaire (see attachment D) consists of:

- Questions about the current situation regarding asking consent for residual tissue use. These questions have been developed for this study.
- Questions to assess which consent procedure clinicians and nurses prefer and their views on the potential drawbacks and advantages (time pressure, properly informed patients) of each of the consent procedures. These questions have been developed for this study.

The post-intervention questionnaire (see attachment E) consists of:

- Questions about how members of the medical staff experienced the three consent procedures with respect to e.g. time pressure and administrative loads. These questions have been developed for this study.
- Questions about how consent procedures in which patients are actively informed about residual tissue use could best be implemented into clinical practice (e.g. timing of the information and who would be able to inform patients). These questions have been developed for this study.

All questionnaires in this study will be tested in a small pilot study. Minor modifications may be implemented based on the results of these studies.

Sample size, statistical power

Sample size is based on an expected response of 75%, allowing sufficient power to address the major outcomes, i.e. consent rates and preferences for procedures, in different subgroups of e.g. hospitals, age and gender. In order to test the hypothesis that the two interventions result in a difference of at least 10% in consent rates, 0.1% non-consent in the opt-out plus or opt-out group versus 10% in the informed consent group, 121 patients per group are needed (80% power and a 2-sided alpha of 0.05; with continuity correction). The difference of 10% is based on our earlier research and the difference is considered a relevant loss of patient materials for research which may lead to selection bias. Because the setting of diagnosis and treatment is different between surgical and outpatient settings and also between general and academic hospitals, it is important to be able to compare the responses in these subgroups. We have also shown in our earlier studies that educational level is associated with preference for a certain procedure and this may also drive response rates of patients. Therefore we propose to include in each intervention group up to 600 patients to be able to conduct subgroup analysis and include adjustment factors; with a response rate of 75% and hence with 450 participants per group we could stratify for 3 to 4 factors, i.e. 90-150 per subgroup, and still have enough power to investigate a difference in response rate of 0.1% versus 10%. Patients will not be stratified by education or sex; we expect these two factors to be equally distributed across groups.

Another reason to obtain a large sample size is that the percentage of non-consenters is small, while it is important to assess their opinions and reasons for non-consent. In the earlier intervention study, 4.6% of respondents indicated in the questionnaire that they wanted to withhold consent or that they did not know whether they wanted to. A sample size of 1800 patients and a response rate of 75% will lead to approximately 62 patients who want to withhold consent or are not sure which choice to make.

A sample size of 450 (based on a conservative estimate of a response rate of 75%) will suffice to test the hypothesis that patients prefer the opt-out plus procedure over the informed consent procedure by a difference of at least 10%, based on the difference found in our earlier studies, i.e. 43% versus 34% (75% power and a 2-sided alpha; with continuity correction). In addition, the sample size will be sufficient to test the hypothesis that patients in the opt-out plus procedure are more satisfied with the procedure they are subjected to than patients in the informed consent procedure. In our earlier research 82% of the patients in the informed consent procedure compared with 65% in opt-out plus procedure indicated that they were satisfied with the procedure.

Basic plan of analysis

Analysis will first be performed to evaluate the comparability of the intervention groups and the control group at study entry in terms of sociodemographic and clinical characteristics. Student’s t-test or appropriate non-parametric statistics will be used, depending on the level of measurement. If the groups are found not to be comparable on one or more background variables, those variables will be employed routinely as covariates in subsequent analyses using multivariate models, such as logistic regression.

The proportion of the outcomes in each intervention arm, i.e. consent rates and patients’ satisfaction with and preference for a consent procedure, the proportion of patients aware of storage of tissue and research use, and the satisfaction with and preference for a consent procedure of the medical staff, will be evaluated by comparison of the informed consent

versus the opt-out plus group, and the opt-out plus versus the opt-out group by non-parametric or parametric tests as appropriate. Patient characteristics, satisfaction with and preferences for the information and consent procedures will be evaluated according to patient characteristics such as age, educational level, sex, hospital type, type of clinical procedure, and disease type. All analyses will be performed using SPSS.

A qualitative analysis of the information needs and appreciation of the patients for the consent procedures will be evaluated through questions in the questionnaire and telephone interviews with a representative group of patients who completed the questionnaire.

Privacy

Data will be coded according to the standard coding rules of the Division of Psychosocial Research and Epidemiology of the NKI-AVL. The principal investigator (Dr. ir. M. K. Schmidt) and study coordinator (Dr. S. Rebers) will have access to the code. The principal investigator will safeguard the key to the code. Our privacy procedure will comply with the 'code goed gedrag'of the Federa.

Publication policy

The results will be published in national and international peer-reviewed journals. If applicable, we will publish in the national press in collaboration with the public relations departments of all participating hospitals. Further, if a consent procedure can be developed in accordance with patients' wishes and research needs, we will develop a brochure about secondary tissue use together with the relevant stakeholders, that can be used in this procedure.

Burden for the patients / respondents

Usual care is offered to patients in the control group. In both intervention groups patients receive brief verbal information and a leaflet, after which they are either asked to sign a consent form or to sign an opt-out form if the patients wants to opt-out. The procedures in the two intervention arms bear no harm to patients. Six weeks after the intervention, patients are asked to fill in a questionnaire. This will take about 45 minutes. 25% of the respondents will be interviewed by telephone, which will take about 20 minutes. Respondents will be sent a second questionnaire, which will take about 10 minutes to fill in. Based on our previous experiences, it is unlikely that the intervention procedures, filling in the questionnaires, or participating in the interview will cause any distress for respondents.

List of attachments

A: Detailed information on the background of this study and on previous research

B: First questionnaire

C: Second questionnaire

D: Pre-intervention questionnaire medical staff

E: Post-intervention questionnaire medical staff

F: Brochure opt-out plus intervention arm

G: Brochure informed consent intervention arm

H: Informationleaflet Tissue Issue

I: Consent form Tissue Issue

J: Patient letter 1

K: Patient letter 2

L: Patient reminder 1

M: Patient reminder 2

N: Inserted sheet with pictures of brochures

O: Checklist medical staff informed consent

P: Checklist medical staff opt-out plus

Q: Checklist medical staff opt-out

Figures

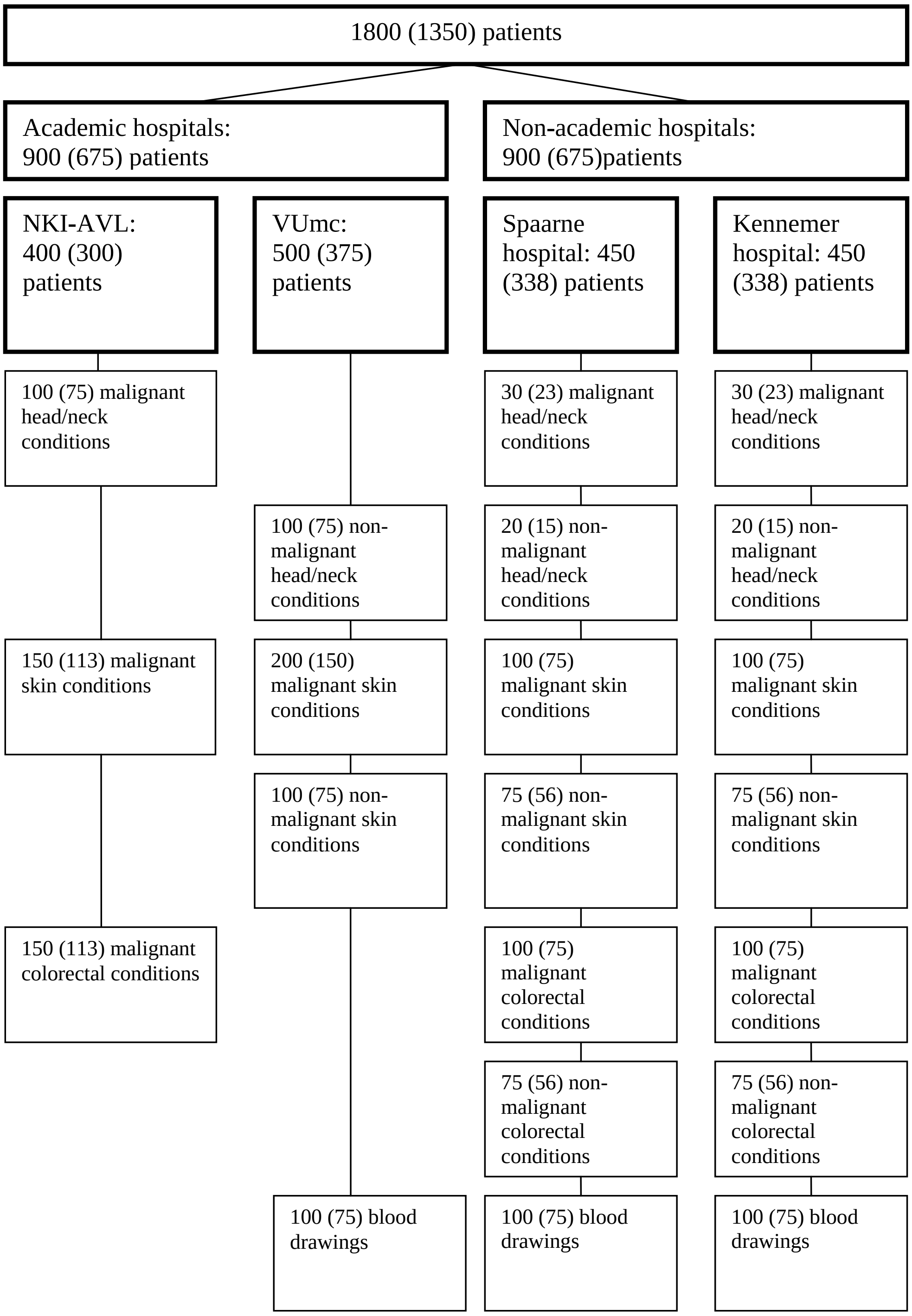

Figure 1: Number of included patients per hospital and condition. Numbers between brackets indicate the expected number of respondents based on a response rate of 75%.

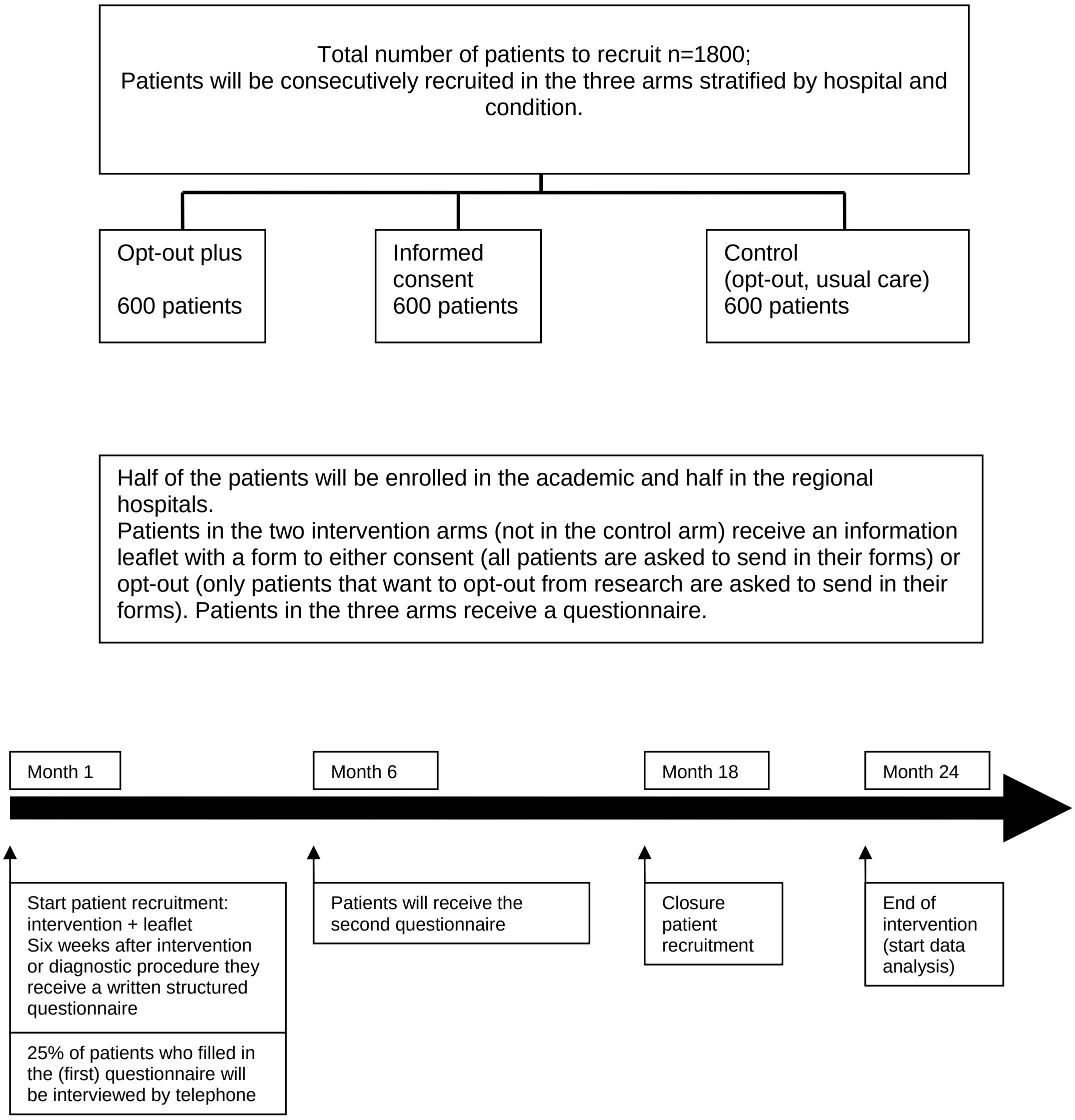

Figure 2: Scheme of the intervention

References

1. Oosterhuis, J. W., Coebergh, J. W. & Van Veen, E.-B. Tumour banks: well-guarded treasures in the interest of patients. Nature Reviews Cancer 3, 73-77. 2003.
Ref Type: Generic

2. Paik, S. *et al.* A multigene assay to predict recurrence of tamoxifen-treated, node-negative breast cancer. New England Journal of Medicine 351(27), 2817-2826. 2004.
Ref Type: Generic

3. Van 't Veer, L. J. *et al.* Gene expression profiling predicts clinical outcome of breast cancer. Nature 351(27), 530-536. 2002.
Ref Type: Generic

4. Maschke, K. J. Navigating an ethical patchwork - human gene banks. Nature Biotechnology 23, 539-545. 2005.
Ref Type: Generic

5. Coebergh, J. W., van Veen, E. B., Vandenbroucke, J. P., Van Diest, P. J. & Oosterhuis, W. One-time general consent for research on biological samples: opt out system for patients is optimal and endorsed in many countries. British Medical Journal 332(7542), 665. 2006.
Ref Type: Generic

6. Van Diest, P. J. No consent should be needed for using leftover body material for scientific purposes. For. British Medical Journal 325(7365), 648-651. 2002.
Ref Type: Generic

7. Furness, P. Consent to using human tissue. *BMJ.* **327**, 759-760 (2003).

8. Bryant, R. J. *et al.* Ownership and uses of human tissue: what are the opinions of surgical in-patients? *J Clin Pathol.* **61**, 322-326 (2008).

9. Wendler, D. One-time general consent for research on biological samples. British Medical Journal 331(7522), 940-944. 2006.
Ref Type: Generic

10. Pentz, R. D., Billot, L. & Wendler, D. Research on stored biological samples: views of African American and White American cancer patients. American Journal of Medical Genetics part A 140(7), 733-739. 2006.
Ref Type: Generic

11. Hansson, M. G., Dillner, J., Bartram, C. R., Carlson, J. A. & Helgesson, G. Should donors be allowed to give broad consent to future biobank research? The Lancet Oncology 7, 266-269. 2006.
Ref Type: Generic

Formatted: English (U.K.)

12. van Veen, E. B. *et al.* TuBaFrost 3: regulatory and ethical issues on the exchange of residual tissue for research across Europe. *Eur J Cancer.* **42**, 2914-2923 (2006).

13. Federation of Medical Scientific Societies. Code for Proper Secondary Use of Human Tissue in the Netherlands (http://www.federa.org/?s=1&m=78&p=&v=v). 2002.
Ref Type: Generic

14. Gevers, J. K. M. Het gebruik van afgenomen lichaamsmateriaal in epidemiologisch onderzoek. Nederlands Tijdschrift voor Geneeskunde 133(4), 173-175. 1989.
Ref Type: Generic

15. Olsthoorn-Heim, E. T. M. Het dossier lichaamsmateriaal. Tijdschrift voor Gezondheidsrecht 5, 306-313. 2003.
Ref Type: Generic

16. Koninklijke Nederlandse Akademie van Wetenschappen.Verkenningscommissie Multifactoriële aandoeningen *Multifactoriële aandoeningen in het genomics-tijdperk.* (2006).

17. Junghans, C., Feder, G., Hemingway, H., Timmis, A. & Jones, M. Recruiting patients to medical research: double blind randomised trial of "opt-in" versus "opt-out" strategies. British Medical Journal 331. 2005.
Ref Type: Generic

18. Wheeler, J., Agarwal, M., Sugden, J., Bladon, M. & Quirke, P. Experiences from the front-line routine consenting of surplus surgically removed tissue: without investment by the National Health Service fully informed consent for all is not available. Journal of Clinical Pathology 60, 351-354. 2007.
Ref Type: Generic

19. Advisory Council on Health Research *Translational Research in the Netherlands. Between Laboratory and Clinical Practice*. (2007).

20. Johnsson, L., Hansson, M. G., Eriksson, S. & Helgesson, G. Patients' refusal to consent to storage and use of samples in Swedish biobanks: cross sectional study. *BMJ* **337**, a345 (2008).

21. Vermeulen, E., Schmidt, M. K., Aaronson, N. K., Kuenen, M. & Van Leeuwen, F. E. Obtaining 'fresh' consent for genetic research with biological samples archived 10 years ago. European Journal of Cancer 45, 1168-1174. 2009.
Ref Type: Generic

22. Vermeulen, E. *et al.* Opt-out plus, the patients' choice: preferences of cancer patients concerning information and consent regimen for future research with biological samples archived in the context of treatment. Journal of Clinical Pathology 62, 275-278. 2009.
Ref Type: Generic

23. Vermeulen, E. *et al.* A trial of consent procedures for future research with clinically derived biological samples. British Journal of Cancer 101, 1505-1512. 2009.
Ref Type: Generic

24. Stegmayr, B. & Asplund, K. Informed consent for genetic research on blood stored for more than a decade: a population based study. *BMJ.* **325**, 634-635 (2002).

25. Kettis-Lindblad, A., Ring, L., Viberth, E. & Hansson, M. G. Genetic research and donation of tissue samples to biobanks. What do potential sample donors in the Swedish general public think? *Eur J Public Health.* **16**, 433-440 (2006).

26. Hoeyer, K. 'Science is really needed--that's all I know': informed consent and the non-verbal practices of collecting blood for genetic research in northern Sweden. *New Genet Soc.* **22**, 229-244 (2003).

27. Kettis-Lindblad, A., Ring, L., Viberth, E. & Hansson, M. G. Perceptions of potential donors in the Swedish public towards information and consent procedures in relation to use of human tissue samples in biobanks: a population-based study. *Scand J Public Health.* **35**, 148-156 (2007).

28. Start, R. D. *et al.* Ownership and uses of human tissue: does the Nuffield bioethics report accord with opinion of surgical inpatients? *BMJ.* **313**, 1366-1368 (1996).

29. Miller, S. M. Monitoring versus blunting styles of coping with cancer influence the information patients want and need about their disease. Cancer 76(2), 167-177. 1995.
Ref Type: Generic

30. Arora, N. K. & McHorney, C. A. Patient preferences for medical decision making: Who really wants to participate? Medical Care 38(3), 335-341. 2000.
Ref Type: Generic

31. Kraetschmer, N., Sharpe, N., Urowitz, S. & Deber, R. B. How does trust affect patient preferences for participation in decision-making? Health Expectations 7, 317-326. 2004.
Ref Type: Generic

32. Geesink, I. & Steegers, C. *Nader gebruik nader onderzocht. Zeggenschap over lichaamsmateriaal*. Rathenau Instituut; TA rapport 0901, Den Haag (2009).

33. Ware, J. E., Kosinkski, M. & Keller, S. D. How to score the SF-12 physical and mental health summary scales. 1998. Lincoln, RI, QualityMetric Incorporated.
Ref Type: Generic

34. Horowitz, M., Wilner, N. & Alvarez, W. Impact of event scale: a measure of subjective stress. Psychosomatic Medicine 41(3), 209-218. 1979.
Ref Type: Generic

35. Brom, D. & Kleber, R. J. De Schok Verwerkings Lijst. Nederlands Tijdschrift voor de Psychologie 40, 164-168. 1985.
Ref Type: Generic

36. Van der Ploeg, E., Mooren, T. M., Kleber, R. J., Van der Velden, P. G. & Brom, D. Construct validation of the Dutch version of the impact of event scale. Psychological Assessment 16(1), 16-26. 2004.
Ref Type: Generic

37. Hagedoorn, M. *et al.* Structure and reliability of Ware's patient satisfaction questionnaire III. Medical Care 41(2), 254-263. 2003.
Ref Type: Generic

38. Van Zuuren, F. J., De Groot, K. I., Mulder, N. L. & Muris, P. Coping with medical threat: An evaluation of the threatening medical situations inventory (TMSI). Personality and Individual Differences 21(1), 21-31. 1996.
Ref Type: Generic

39. Van Zuuren, F. J. & Hanewald, G. J. F. P. Cognitieve toenadering en vermijding in medisch bedreigende situaties: de ontwikkeling van een vragenlijst. Gedragstherapie 26(1), 33-48. 1993.
Ref Type: Generic
